# Therapeutic potential of exerkines in neurodegenerative and mental disorders: a narrative review

**DOI:** 10.3389/fphys.2026.1793043

**Published:** 2026-03-19

**Authors:** Suwol Yang, Hye-Won Sang, Seoyeon Kim, Eun-Jeong Cho, Youngju Choi, Dong Woo Kang, Young C. Jang, Dong-Ho Park, Hyo-Bum Kwak, Jang Soo Yook

**Affiliations:** 1 Department of Biomedical Science & Engineering, Inha University, Incheon, Republic of Korea; 2 Institute of Sports and Arts Convergence, Inha University, Incheon, Republic of Korea; 3 College of Medicine, Seoul St. Mary’s Hospital, The Catholic University of Korea, Seoul, Republic of Korea; 4 Wallace H. Coulter Department of Biomedical Engineering, Georgia Institute of Technology and Emory University School of Medicine, Atlanta, GA, United States; 5 Department of Orthopedics, Emory Musculoskeletal Institute, Emory School of Medicine, Atlanta, GA, United States; 6 Department of Kinesiology, Inha University, Incheon, Republic of Korea; 7 Division of Humanities and Social Sciences, Pohang University of Science and Technology (POSTECH), Pohang, Republic of Korea; 8 Graduate School of Convergence Science and Technology, Pohang University of Science and Technology (POSTECH), Pohang, Republic of Korea

**Keywords:** exerkines, aerobic exercise, resistance exercise, muscle-brain crosstalk, blood–brain barrier, Alzheimer’s disease, Parkinson’s disease, mental disorders

## Abstract

Neurodegenerative and mental disorders impose significant global disease burdens and pose serious social and economic challenges. Physical exercise (PE) exerts beneficial effects on brain health, contributing to a reduction in the risk of Alzheimer’s disease (AD), Parkinson’s disease (PD), depression, anxiety, and post-traumatic stress disorder (PTSD). To understand these effects of PE, a variety of molecules released from various tissues in response to PE have been discovered, which are collectively called ‘exerkines’. In particular, the skeletal muscle acts as an endocrine organ, secreting exerkines and is included in the category of myokines that facilitate direct or indirect crosstalk between the muscle and the brain. Although muscles actively interact with organs such as the liver, pancreas, and adipose tissue, the precise mechanisms of muscle–brain communication have yet to be fully elucidated. In the skeletal muscle, the types of exerkines secreted and their effects vary depending on the PE modality. Furthermore, these exerkines can cross the blood-brain barrier (BBB) to exert direct effects or act indirectly *via* molecular signaling pathways, contributing to the modulation of the brain microenvironment, attenuation of neuroinflammation, and neurodegeneration. Previous studies have indicated that brain-derived neurotrophic factor (BDNF), irisin, cathepsin B (CTSB), interleukin-6 (IL-6), and insulin-like growth factor 1 (IGF-1) are involved in enhancing cognitive performance and improving behavioral outcomes by promoting neurogenesis and synaptic plasticity. This review comprehensively discusses the effects of exerkines on the brain and the physiological responses manifested in neurodegenerative and mental disorders focusing primarily on findings from rodent models. Based on these insights, this review proposes future research directions to translate preclinical findings into therapeutic strategies.

## 1 Introduction

Neurodegenerative and psychiatric disorders represent some of the most significant global health challenges and, impose a substantial burdens on individuals, healthcare systems, and society (Mitchell et al., 2025). In particular, the increasing incidence of Alzheimer’s disease (AD), Parkinson’s disease (PD), and mental disorders such as depression, anxiety, and post-traumatic stress disorder (PTSD) is closely linked to disability and mortality, especially in the elderly population (Collaborators, 2021, Collaborators, 2024; Mao et al., 2025). Despite the urgency posed by an aging global population, effective treatments remain elusive as existing pharmacological interventions often present significant side effects and limited efficacy (Alzheimer’s Association, 2025; Bloem et al., 2021). Although the development of fundamental therapeutics is crucial, the most viable strategies for aging populations should focus on delaying disease progression and preventing disease onset.

Physical exercise (PE) is considered a potent intervention capable of mitigating many modifiable risk factors associated with neurodegenerative diseases and psychiatric disorders (Lee et al., 2023; Nagamatsu et al., 2014). Consistent with this perspective, accumulating clinical evidence demonstrates that regular PE improves cognitive function, reduces the risk of dementia, and alleviates the symptoms of psychiatric conditions such as depression and anxiety (Cámara-Calmaestra et al., 2022; Goodwin et al., 2008; López-Ortiz et al., 2023; Zhen et al., 2022). These beneficial effects are associated with enhanced neuroplasticity, increased hippocampal neurogenesis, and improved synaptic transmission (Campos et al., 2023; da Rocha et al., 2025; Guo et al., 2022). Despite these benefits, the precise molecular mechanisms through which PE induces structural and functional changes in the central nervous system (CNS) remain unclear.

**Figure 1:**
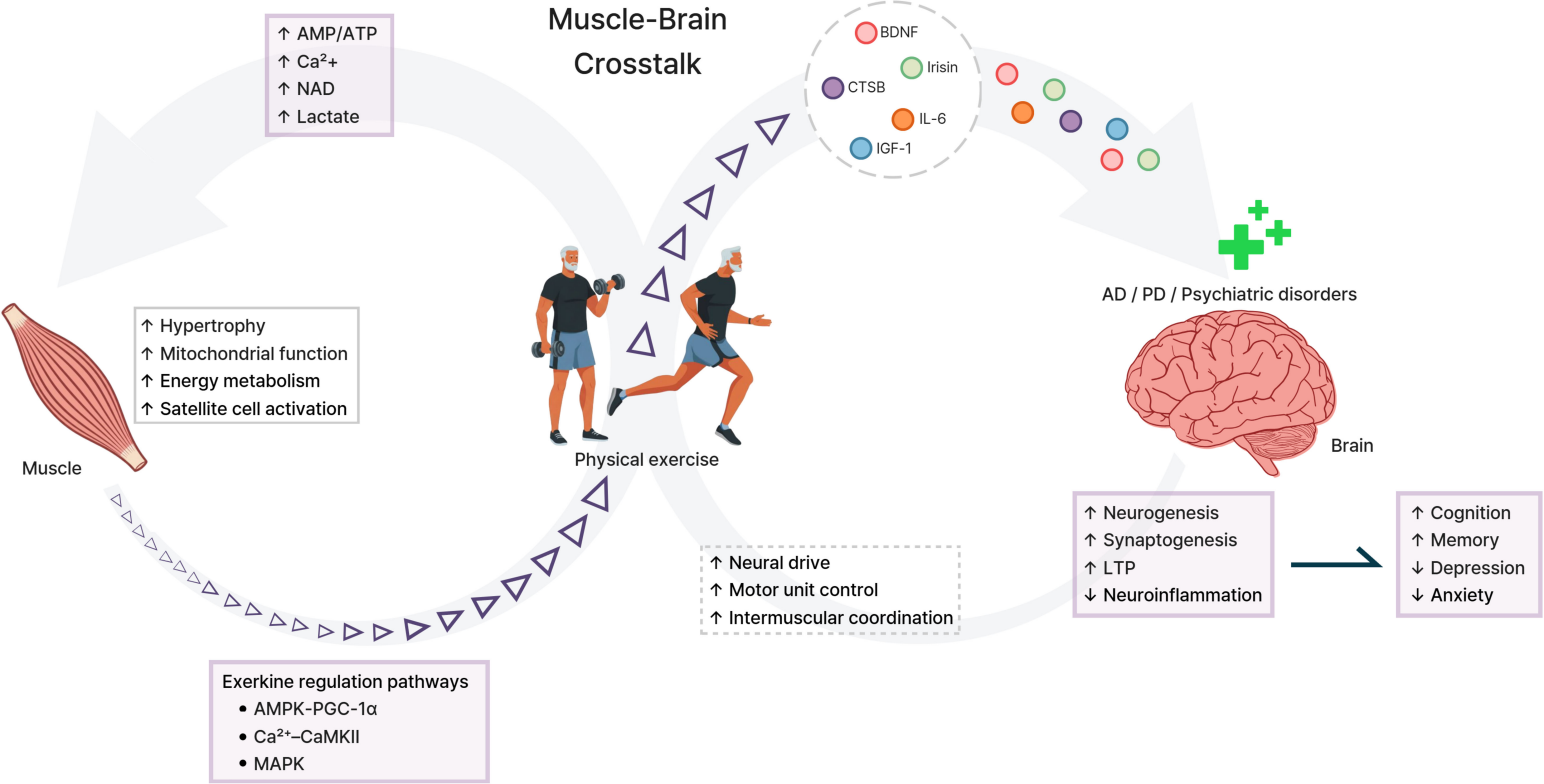
Schematic representation of physical exercise–induced muscle–brain crosstalk. Physical exercise activates intracellular signaling pathways in skeletal muscle (e.g., AMPK–PGC-1α, Ca^2+^–CaMKII, and MAPK), driving metabolic adaptations and the release of exercise-responsive exerkines and growth factors (e.g., irisin/FNDC5, CTSB, IL-6, and IGF-1, and exercise-associated BDNF). These circulating mediators reach the brain and signal across the blood–brain barrier (BBB) *via* humoral and neurovascular routes, promoting neurogenesis, synaptogenesis, and long-term potentiation (LTP) while attenuating neuroinflammation. Collectively, these mechanisms may improve cognition and mental health, highlighting the therapeutic potential of exercise in neurodegenerative diseases (AD, PD) and psychiatric disorders. AD, Alzheimer’s disease; PD, Parkinson’s disease; AMPK, AMP-activated protein kinase; PGC-1α, peroxisome proliferator-activated receptor gamma coactivator 1-alpha; CaMKII, Ca^2+^/calmodulin-dependent protein kinase II; MAPK, mitogen-activated protein kinase; BDNF, brain-derived neurotrophic factor; CTSB, cathepsin B; IL-6, interleukin-6; IGF-1, insulin-like growth factor 1; LTP, long-term potentiation; BBB, blood–brain barrier.

To understand the physiological benefits of PE for brain health, several signaling molecules released from diverse tissues in response to PE have been identified. These secreted factors are collectively termed “exerkines” (Chow et al., 2022; Safdar et al., 2016). Among these factors, some exerkines are secreted from skeletal muscle in an activity- and context-dependent manner and play crucial roles in muscle–brain crosstalk (Chow et al., 2022; Pedersen, 2019). Their activity increases in a contraction dependent manner during PE (Fukada and Nakamura, 2021; Pedersen, 2023), while also exerting local autocrine and paracrine effects that support muscle metabolic homeostasis (Pedersen, 2019). Secretion profiles vary with PE modality (Severinsen and Pedersen, 2020) because different demands recruit different fiber types. Aerobic exercise (AE) engages oxidative type I fibers and induces metabolic stress, whereas resistance exercise (RE) recruits glycolytic type II fibers under mechanical tension (Bawa et al., 2014; Qaisar et al., 2016). Therefore, PE type influences the secretome, while PE intensity and duration further modulate its composition and overall magnitude (Hughes et al., 2018; Leuchtmann et al., 2021). However, integrated studies that systematically compare secretion patterns across PE types remain limited. Circulating signals can influence CNS function, making them promising biomarkers and targets (Pedersen, 2019). Fibronectin type III domain-containing protein 5 (FNDC5)/irisin, brain-derived neurotrophic factor (BDNF), cathepsin B (CTSB), interleukin-6 (IL-6), and insulin-like growth factor 1 (IGF-1) may affect AD and PD via neuroinflammation, energy metabolism, and hippocampal plasticity (**Figure 1**) (Lee et al., 2021), and may be related to depression, anxiety, and PTSD through BDNF-dependent neuroplasticity, oxidative stress, and hypothalamic–pituitary–adrenal (HPA) axis regulation (Montgomery and Grant, 2026).

Previous reviews have primarily focused on the neuroprotective and therapeutic effects of exerkines within the CNS and the underlying mechanisms (Pedersen, 2019; Rai and Demontis, 2022; Wrann, 2015). In contrast, secretion mechanisms and regulatory functions in the skeletal muscles have received relatively limited attention. For leading candidates such as irisin and CTSB, it remains debated whether muscle contraction reliably drives their release into the circulation (Sugimoto et al., 2022). Moreover, integrated comparative studies that characterize secretion patterns across levels of PE intensity and duration remain scarce. Regarding central delivery, blood-brain barrier (BBB) permeability and transport pathways likely involve both direct and indirect routes, but key uncertainties and controversies remain (Maak et al., 2021; Rai and Demontis, 2022). Finally, research has focused relatively more on neurodegenerative disorders, whereas evidence in psychiatric conditions remains limited (Liu et al., 2024; Mojtabavi et al., 2020).

The primary aim of this review is to provide a comprehensive overview of the biogenesis and secretion mechanisms of exerkines that may mediate muscle–brain crosstalk, and to evaluate their potential therapeutic roles in neurodegenerative and mental disorders. First, we systematically summarized prior evidence on the key exerkines implicated in brain function, focusing on their production pathways, secretion mechanisms, and physiological functions in target organs. To strengthen the molecular rationale for prescribing PE and to inform strategies for optimizing exerkine release, we compared exerkine secretion profiles by PE modality (AE vs. RE) and categorized them accordingly (**Table 1**). We then examined the current evidence and ongoing debates on whether, and through what direct or indirect routes, exerkines reach the brain, and discussed the possibility that they may help restore BBB–mediated crosstalk disrupted by brain disorders. Finally, drawing on recent studies, particularly those using well-controlled preclinical rodent models with clearly defined pathological phenotypes, we integrated evidence for exerkine-mediated neuroprotective mechanisms across a broad spectrum of diseases, including AD, PD, depression, anxiety, and PTSD (**Table 2**). This review proposes a conceptual framework to advance the clinical translation of exerkines as therapeutic mediators.

**Table 1:** Exercise modality–specific exerkine response patterns.

Exerkine	Primary driver	Aerobic exercise (AE)	Resistance exercise (RE)
Irisin/FNDC5	intensity, sampling timing	► [Muscle] HIIT > MICT (overweight/obese adolescents) (Archundia-Herrera et al., 2017) ► [Blood] Isoenergetic high-intensity AE: ↑circulating irisin (Tsuchiya et al., 2014)	► [Blood] Peak ~1 h post-acute RE (Tsuchiya et al., 2015) ► [Blood vs Muscle] ↑circulating irisin despite unchanged muscle FNDC5 mRNA (Nygaard et al., 2015) ► [Blood] ↑basal irisin after 12-week RE in older adults (Kim et al., 2015)
BDNF	muscle mass, total work	► [Blood] High-intensity AE: ↑circulating BDNF (Cho et al., 2012) ► [Blood] Rapid decline toward baseline in early recovery (Heyman et al., 2012)	► [Blood] Hypertrophy-style (short rest/high volume)> strength-style (Marston et al., 2017) ► [Blood] No increase in plasma BDNF after acute RE (Correia et al., 2010)
CTSB	protocol, timing, training history	► [Blood] High-intensity AE (high %VO₂max): ↑CTSB (Mazo et al., 2022) ► [Blood] Chronic AE: ↑resting CTSB (Gaitan et al., 2021) ► [Blood] 6-week HIIT: no significant CTSB change (Nicolini et al., 2019) ► [Blood] Lower resting CTSB in endurance-trained (De la Rosa et al., 2019)	► [Blood] No early post-RE CTSB change after ~80% 1RM (~80% 1RM) (Johnson et al., 2020) ► [Blood] ↑resting serum CTSB after 12-week RE in obese women (Sung et al., 2017)
IL-6	duration, fuel stress	► [Blood] Marked increase during prolonged endurance AE (Steensberg et al., 2000) ► [Blood & Muscle] Attenuated increase with carbohydrate intake (Nieman et al., 2003) ► [Blood & Muscle] Amplified response under low glycogen (Keller et al., 2001) ► [Muscle] Blunted IL-6 mRNA response after endurance training (Fischer et al., 2004)	► [Blood & Muscle] Acute ↑muscle IL-6 mRNA, with ↑circulating IL-6 mainly under high metabolic stress (Annibalini et al., 2019) ► [Blood] Unchanged basal IL-6 after chronic resistance training (Libardi et al., 2012)
IGF-1	context (intensity/duration), age	► [Blood] Intensity-dependent acute ↑IGF-1 (Schwarz et al., 1996) ► [Blood] ↓circulating IGF-1 after prolonged endurance (Copeland and Verzosa, 2014) ► [Blood] ↑circulating IGF-1 (acute, low-volume sprint interval cycling) (Cui et al., 2015)	► [Blood] No IGF-I change; volume-dependent ↑IGFBP-1 (Nindl et al., 2009) ► [Muscle] ↑IGF-I mRNA ~48 h post high-load RE (eccentric > concentric) (Bamman et al., 2001)
CX3CL1 (fractalkine)	sampling timing, intensity	► [Blood & Muscle] Acute 1-h one-legged endurance exercise ↑CX3CL1 mRNA (Catoire et al., 2014) ► [Blood & Muscle] No change in CX3CL1 after 6-month aerobic training (Verheggen et al., 2016) ► [Muscle] Acute 1-h cycling ↑CX3CL1 mRNA and protein levels; vastus lateralis (Stromberg et al., 2016)	► [Muscle] Acute resistance exercise ↑CX3CL1 mRNA (Della Gatta et al., 2014) ► [Blood] Low-intensity resistance exercise program ↑serum CX3CL1 (Hashida et al., 2021)
MCP-1 (CCL2)	inflammatory, metabolic stress, duration	► [Blood & Muscle] Acute 1-h one-legged endurance exercise ↑MCP-1 mRNA and protein levels (Catoire et al., 2014) ► [Muscle & Blood] Acute 45-min cycling ↑MCP-1 mRNA (Balan et al., 2021) ► [Blood] 2-week moderate-intensity endurance training (MIT) ↑MCP-1 (Middelbeek et al., 2021)	► [Muscle] Acute isokinetic exercise ↑muscle MCP-1 expression (Della Gatta et al., 2014) ► [Blood] 12-week resistance training ↓circulating MCP-1 in older adults (Oliveira et al., 2020)

**Table 2:** Summary of preclinical rodent studies on exerkine regulation and therapeutic outcomes in neurodegenerative and mental disorder.

Animal model			Exercise		Effects of exercise on exerkines			Related outcomes	Reference
Phenotype	Sex	Age	Type	Protocol	Blood	Muscle	Brain		
5xFAD (AD)	Male	6 wk	Voluntary wheel running (AE)	6 mo 30 ± 1 km/week ~20 ± 1 h/week	—	—	[Hippocampus] ↑ BDNF	↓ Cognitive impairment ↑ Spatial learning & Memory	Belaya et al., 2020
3xTg (AD)	Male	9 mo	Resistance ladder climbing (RE)	4 wk (alternate days) 15 reps/session (2 min rest)	= IL-6	—	[Hippocampus] [F. cortex] ↑ *IL-6* mRNA	↓ Neuroinflammation ↓ Cognitive impairment ↓ Aβ plaques & Tau ↑ Learning & Memory	Liu et al., 2020
3xTg (AD)	Female	3 mo	Treadmill running (AE)	9 wk 5 d/wk 15 m/min 30→90 min	—	[Gastrocnemius] = CTSB	[Hippocampus] ↑ IGF-1 = BDNF	↑ Motor performance	Pena et al., 2020
			Weighted ladder climbing (50→100% BW) (RE)	9 wk 3 sessions/wk 16→10 reps/session (1 min rest)	—			↓ Hippocampal Aβ ↑ Motor performance ↑ Grip strength	
i.c.v. AβO model (AD)	Male	2.5-3 mo	swimming (AE)	5 wk 5 d/wk 1 h/day	—	[Gastrocnemius] = *Fndc5*	[Hippocampus] ↑ Irisin/FNDC5 (mRNA·protein) ↑ BDNF	↑ Cognitive & Memory function ↑ Synaptic plasticity	Lourenco et al., 2019
3xTg (AD)	Male	2.5 mo	Forced wheel running (AE)	12 wk 1-3 d/wk 60 min/d 8 m/min	[Serum] ↓ MCP-1	—	[Cortex] = MCP-1	—	Haskins et al., 2016
MPTP mouse (PD)	Male	6-8 wk	Treadmill running (AE)	10 wk 5 d/wk 30 min/session x 2 12 m/min	—	—	[Hippocampus] ↑ FNDC5 ↑ BDNF	↓ Depression-like behavior ↑ Learning & Memory ↑ Motor performance ↑ DA neuron integrity ↑ Synaptic structure	Shan et al., 2025
MPTP mouse (PD)	Male	8 wk	Treadmill running (AE)	10 wk 5 d/wk 10→60 min/d 8→12 m/min	—	—	[Hippocampus] ↑ Irisin/FNDC5	↓ α-synuclein ↓ Neuroinflammation ↑ Motor function ↑ TH+ cells & AHN ↑ Memory	Zhao et al., 2025
MPTP mouse (3-8 wk i.p.) (PD)	Male	7 wk	Treadmill running (AE)	8 wk 5 d/wk 60 min/d 12 m/min	[Serum] ↑ FNDC5	[Quadriceps] ↑ FNDC5	[Whole brain] ↑ FNDC5	↑ Cognitive & Memory function ↑ Motor function ↑ TH+ cells & DA	Tang et al., 2023
MPTP mouse (PD)	Male	2 mo	Treadmill running (AE)	10 wk 5 d/wk 10→60 min/d 8→12 m/min	[Serum] ↑ Irisin	—	[SN] ↑ Irisin	↓ Neuroinflammation ↑ TH+ cells ↑ Motor function	Wang et al., 2025
CUMS rat (depression)	Male	Adult	Treadmill running (AE)	8 wk 45 min 1.02 km/h	—	—	[Hippocampus] ↑ *Bdnf* mRNA ↑ IGF-1 ↑ FNDC5	↓ Hippocampal apoptosis ↑ Synaptic plasticity ↓ Depression	Kang et al., 2020
			Weight-bearing treadmill (interval) (RE)	8 wk 20 cycles/d 1.02 km/h (30% BW load)			↑ *IGF-1* mRNA ↑ IGF-1 ↑ IGF-1R		
CUS rat (depression)	Male	2 mo	Continuous treadmill running (AE)	6 wk 5 d/wk 22-42 min 23-27 m/min	—	—	[Hippocampus] ↑ BDNF ↑ FNDC5 (HIIT > Con)	↓ Anxiety ↓Depression	Babaei et al., 2021
			HIIT (RE)	6 wk 5 d/wk 38-42 m/min x (2-6) (2 min rest)					
CUS rat (depression)	Male	6-8 wk	Treadmill running (AE)	6 wk 5 d/wk 20 min/d	—	—	[Hippocampus] ↓ IL-6	↓ Depression ↓ Neuroinflammation (IL-1β)	Xiao et al., 2021
SPS rat + OVX factorial (PTSD)	Female	Adult	Forced running wheel (FRW) (AE)	4 wk 5 d/wk 30 min/d 10 m/min	—	—	[Hippocampus] [PFC] ↑ BDNF ↑ IGF-1	↓ Anxiety ↑ Cognitive function	Amiri et al., 2024
SPS rat (PTSD)	Male and female	Adult	Treadmill running (pre-trauma) (AE)	4 wk 5 d/wk 30 min/d 10-15 m/min	[Serum] ↑ BDNF	—	[Hippocampus] ↑ BDNF	↓ Apoptosis ↓ Anxiety	Mirjalili et al., 2022
SPS rat + BMSCs (PTSD)	Male	Adult	Treadmill running (AE)	4 wk 5 d/wk 30 min/d 10 m/min	[Serum] ↑ BDNF ↑ IGF-1	—	[Hippocampus] ↑ IGF-1	↓ Anxiety	Eshaghi-Gorji et al., 2024
[PFC] ↑ IGF-1 ↑ BDNF									

Level of protein/gene expression: ↑ increase; ↓ decrease; = no change. CUMS, chronic unpredictable mild stress; CUS, chronic unpredictable stress; SPS, single prolonged stress; OVX, ovariectomized; HIIT, high-intensity interval training; DA, dopamine; TH, tyrosine hydroxylase; F. cortex, frontal cortex; AβO, amyloid-β oligomers; BW, body weight; SN; substantia nigra; BMSCs, bone marrow mesenchymal stem cells.

## 2 Exerkines: secretion mechanisms and functions

### 2.1 FNDC5/Irisin

Irisin is a exerkine generated by proteolytic cleavage of FNDC5, a type I membrane protein induced in skeletal muscle through the AMP-activated protein kinase (AMPK)/peroxisome proliferator-activated receptor gamma coactivator 1-alpha (PGC-1α) pathway (Boström et al., 2012; Shan et al., 2013). FNDC5 is processed by cleavage of its N-terminal extracellular domain (Boström et al., 2012), but the exact cleavage site and responsible protease(s), including possible a disintegrin and metalloproteinase (ADAM) family involvement (e.g., ADAM10), remain unresolved (Nie and Liu, 2017; Witmer et al., 2024). In humans, upstream ATG-dependent translation initiation, glycosylation, and downstream processing have been proposed, but their sequence and regulation are still unclear (Nie and Liu, 2017; Witmer et al., 2024). Irisin signals largely via αV integrins (e.g., αVβ5) (Kim et al., 2018) and can act endocrinologically as cargo within extracellular vesicles (EVs). PE increases the release of small EVs enriched in FNDC5/irisin. Likewise, muscle-specific FNDC5 rescue increases circulating FNDC5/irisin-rich EVs, supporting EV-mediated systemic delivery as a key endocrine route (Chi et al., 2022; Shi et al., 2024; Whitham et al., 2018). Systemic irisin promotes osteogenesis and reduces adiposity (Boström et al., 2012; Colaianni et al., 2015; Kim et al., 2018). It also enhances satellite cell function, mitochondrial biogenesis, and glucose uptake (Lee et al., 2015, Lee et al., 2019; Reza et al., 2017; Vaughan et al., 2014). In the brain, FNDC5/irisin induces hippocampal *Bdnf* mRNA and adult neurogenesis (Islam et al., 2021; Wrann et al., 2013), attenuates microglial inflammation (Wang et al., 2022), and reduces amyloid-β (Aβ) burden through astrocytic neprilysin release (Kim et al., 2023).

### 2.2 Brain-derived neurotrophic factor

BDNF is a member of the neurotrophin family (NGF), produced mainly by neurons and is also expressed in skeletal muscles (Barde, 2025). The BDNF locus uses multiple promoters to generate diverse 5′ untranslated region (5′-UTR) transcripts that encode the same protein precursor, preproBDNF (proBDNF), which can be stored/secreted as proBDNF or proteolytically processed into mature BDNF (Aid et al., 2007; Greenberg et al., 2009; Pruunsild et al., 2007). Mature BDNF binds to tropomyosin receptor kinase B (TrkB) and activates downstream mitogen-activated protein kinase (MAPK) and phosphoinositide 3-kinase (PI3K)/protein kinase B (Akt) signaling, thereby promoting neuronal survival, synaptic plasticity, and long-term potentiation (LTP), while also contributing to cognitive functions including learning, memory and mood regulation, in stress- and depression-related contexts (Barde, 2025; Duman and Monteggia, 2006; Lu et al., 2014). Impaired BDNF/TrkB signaling and dysregulated BDNF intracellular transport have been implicated in depression and AD, and the pharmacological enhancement of TrkB-dependent plasticity has emerged as a therapeutic strategy (Casarotto et al., 2022; Lu et al., 2014). In skeletal muscles, contraction induces BDNF expression, which mainly acts in an autocrine/paracrine manner to coordinate metabolic adaptation and muscle repair (Matthews et al., 2009). BDNF promotes AMPK-dependent lipid oxidation, mitochondrial quality control (remodeling/mitophagy) under lipid stress, and muscle regeneration *via* satellite cell differentiation (Ahuja et al., 2022; Clow and Jasmin, 2010; Matthews et al., 2009).

### 2.3 Cathepsin B

CTSB is a lysosomal cysteine protease widely expressed across tissues (Turk et al., 2012). PE and metabolic stress have suggested CTSB as a candidate exerkine, with skeletal muscle proposed as a source contributing to increased circulating CTSB levels (Moon et al., 2016). AMPK/PGC-1α activation induces *Ctsb* transcription (Moon et al., 2016). Newly synthesized CTSB undergoes endoplasmic reticulum (ER) entry/processing, Golgi glycosylation, and proteolytic maturation in acidic endolysosomal compartments (Xie et al., 2023). In muscle, CTSB has been shown to act in an autocrine manner to support myogenesis by promoting myoblast differentiation and fusion; during fusion, mature CTSB translocates to the cell surface and is released in an active form (Jane et al., 2006). Consistently, CTSB depletion or antisense-mediated knockdown in C2C12 cells impairs their survival and fusion, resulting in fewer and smaller multinucleated myotubes (Gogos et al., 1996). In neurons and glia, lysosomal CTSB supports proteostasis through lysosomal degradation pathways and has been associated with increased doublecortin (DCX) and BDNF (often assessed in the hippocampus) as well as improved cognitive performance (Moon et al., 2016; Ni et al., 2022). Conversely, under pathological conditions, lysosomal membrane permeabilization can lead to cytosolic release of CTSB, triggering inflammatory signaling and promoting cell death (Saudenova et al., 2022; Xie et al., 2023). In microglia, CTSB contributes to interleukin-1 beta (IL-1β) release and can amplify neuroinflammation (Bai et al., 2018; Nakanishi, 2020). PE-induced CTSB may also enter the circulation and influence remote tissues; however, its functions differ across the lysosomal, cytosolic, and extracellular pools, necessitating context-specific interpretation (Brix, 2018).

### 2.4 Interleukin-6

IL-6 is a representative myokine and the exerkine (Febbraio and Pedersen, 2005). Electrical pulse stimulation (EPS)-induced contractile activity activates MAPK signaling to promote *IL-6* transcription (Whitham et al., 2012), after which IL-6 is processed and secreted *via* the conventional ER–Golgi apparatus secretory pathway (Verboogen et al., 2019). IL-6 acts via IL-6 receptor alpha (IL-6Rα) and the shared co-receptor gp130, producing autocrine/paracrine responses that depend on the magnitude and duration of exposure (Rose-John, 2012). Classic signaling requires membrane-bound IL-6Rα, whereas trans-signaling uses soluble IL-6R (sIL-6R) to activate gp130 on IL-6Rα negative cells (Rose-John, 2012). Acutely, IL-6 activates AMPK to enhance glucose and lipid metabolism, and may support hypertrophy by promoting satellite cell proliferation (Guo et al., 2024; Johnson et al., 2023). Systemically, PE-induced IL-6 mediates crosstalk with the liver, adipose tissue, and pancreas to maintain substrate availability and glycemic control (Ellingsgaard et al., 2020; Lin et al., 2023). In chronic conditions such as cancer cachexia, IL-6 has been implicated in muscle wasting (Bonetto et al., 2012). EV-displayed IL-6R may further modulate the target cell responsiveness, suggesting an additional endocrine-like route (Arnold et al., 2020).

In the CNS, IL-6 can exert neurotrophic and neuromodulatory actions that support synaptic plasticity and neurogenesis (Erta et al., 2012; Gruol, 2015). However, chronic IL-6 exposure may downregulate endothelial tight junction proteins (claudin-5 and occludin) and impair cognition (Takata et al., 2021). Thus, IL-6 effects are context-dependent and, differ between transient PE pulses and chronic inflammation (Pedersen and Febbraio, 2008).

### 2.5 Insulin-like growth factor 1

IGF-1 is a systemic endocrine effector of the growth hormone (GH)/IGF axis and a load-responsive exerkine expressed locally in skeletal muscles (Pedersen and Febbraio, 2012). IGF-1 is precisely regulated by the differential (often temporally patterned) induction of distinct isoforms and post-translational mechanisms (Philippou et al., 2014; Zanou and Gailly, 2013). Functionally, IGF-1 drives growth by enhancing protein synthesis via mechanistic target of rapamycin complex 1 (mTORC1) pathway (Schiaffino and Mammucari, 2011) and inhibiting FoxO-dependent ubiquitin–proteasome (UPS) catabolic programs (Sandri et al., 2004; Schiaffino and Mammucari, 2011). Collectively, these effects preserve regenerative capacity in atrophy and aging models, and induce hypertrophy in human myotubes (Musaro et al., 2001; Yoshida et al., 2010). Circulating IGF-1 is predominantly produced by the liver via GH-dependent, signal transducer and activator of transcription 5B (STAT5B)-mediated transcription and circulates largely in an insulin-like growth factor-binding protein 3 (IGFBP-3)/acid-labile subunit (ALS) complex, with target tissue bioavailability governed by IGFBP binding and proteolysis (Baxter, 2024; Rotwein, 2012). A recent study proposed that systemic IGF-1 can be packaged into EVs by choroid plexus epithelial cells, released into the cerebrospinal fluid, and taken up by hippocampal neurons in the immature brain (Ortenlof et al., 2024). In the CNS, IGF-1 activates the PI3K/Akt and MAPK pathways to support neuronal survival, synaptic plasticity, and glia-mediated metabolic adaptation (Fernandez and Torres-Aleman, 2012). PE-induced brain uptake of circulating IGF-1 supports hippocampal neurogenesis and neuroprotection, including reduced Aβ burden and protection of dopaminergic neurons (Carro et al., 2002; Trejo et al., 2001; Wang et al., 2020).

## 3 Exercise type-specific responses and regulation of exerkines

Exerkine responses to AE and RE depend on the intensity, duration, muscle recruitment, fatigue, and sampling timing immediately after PE compared with the early recovery phase, and circulating signals may diverge from intramuscular changes (**Table 1**) (Bettariga et al., 2024; Nygaard et al., 2015). Thus, identical protocols can yield different directions and magnitudes across serum versus plasma, and across sampling time points (Bettariga et al., 2024; Nygaard et al., 2015).

### 3.1 Aerobic exercise

In AE, an intensity-driven cluster of acute responders is often observed first, particularly in terms of irisin and circulating BDNF levels (Archundia-Herrera et al., 2017; Lee and Ko, 2025; Tsuchiya et al., 2014). High-intensity interval training (HIIT) increases skeletal muscle irisin protein levels more than moderate-intensity continuous exercise (Archundia-Herrera et al., 2017), and high-intensity AE robustly elevates circulating irisin (Lee and Ko, 2025; Tsuchiya et al., 2014). In patients with PD, 12 weeks of regular AE increased serum irisin levels, and the magnitude of this increase was associated with improvements in balance function (Berg Balance Scale, BBS) (Zhang et al., 2023). Similarly, circulating BDNF levels exhibit an acute spike immediately after high-intensity or high-volume AE, followed by a rapid decline during early recovery (Cho et al., 2012; Heyman et al., 2012). Meta-analytic evidence indicates that exercise interventions tend to increase plasma BDNF levels in neurodegenerative disorders such as AD and PD (Ruiz-Gonzalez et al., 2021). In major depressive disorder (MDD), exercise training related increases in BDNF have been reported alongside symptom reduction, and changes in BDNF and IL-6 were associated with greater clinical improvement (da Cunha et al., 2023). In contrast, IL-6 levels during AE shows a duration dependent pattern, rising progressively during prolonged exercise with substantial energetic demand, typically after ~60–120 min (Keller et al., 2001; Steensberg et al., 2000). Accordingly, acute, muscle-derived IL-6 levels should be distinguished from chronically elevated basal IL-6 levels (Keller et al., 2001; Steensberg et al., 2000). In PTSD, both trauma-sensitive yoga and cognitive processing therapy reduced PTSD symptoms; however, IL-6 and C-reactive protein (CRP) differed across the arms, underscoring the sensitivity to intervention type and sampling timing (Zaccari et al., 2023). IGF-1 is also context-dependent; brief high-intensity bouts can transiently increase IGF-1 levels, whereas prolonged endurance exercise is often associated with decreases (Copeland and Verzosa, 2014; Cui et al., 2015). Overall, during AE, irisin and BDNF displayed intensity-sensitive, immediate post-exercise peaks, whereas IL-6 was driven primarily by exercise duration and metabolic load.

### 3.2 Resistance exercise

In RE, the primary determinants of exerkine responses are the amount of recruited muscle mass, total work performed (volume), and depth of fatigue (Kraemer and Ratamess, 2005; Marano et al., 2025; Marston et al., 2017). Accordingly, irisin and BDNF often present as transient, pulse-like increases (Marston et al., 2017; Tsuchiya et al., 2015). Irisin has been reported to peak within ~1 hour post-exercise (Tsuchiya et al., 2015), and resting levels may increase after ≥12 weeks of RE (Kim et al., 2015; Zhao et al., 2017). Likewise, BDNF levels tend to increase more prominently after hypertrophy- oriented, high-volume sessions that recruit large muscle groups (Correia et al., 2010; Marston et al., 2017). A similar acute pulse pattern has been observed in anxiety disorders. In panic disorder, a clinical study reported that a 30 min AE bout acutely increased reduced baseline circulating BDNF levels (Ströhle et al., 2010). In contrast, CTSB and IL-6 levels may show limited or inconsistent acute changes in circulating levels after a single RE session (Annibalini et al., 2019; Johnson et al., 2020). Nevertheless, repeated RE may be associated with a higher resting CTSB (Sung et al., 2017), and upregulation of IL-6 mRNA in skeletal muscle has also been reported (Annibalini et al., 2019). IGF-1 adaptations are often more evident locally (in the muscle) than systemically (in the blood), with volume-sensitive changes in IGFBP-1 and delayed, recovery-phase increases in intramuscular IGF-1/mechano growth factor (MGF) mRNA transcripts (Bamman et al., 2001; Nindl et al., 2009). Thus, reliance on circulating IGF-1 alone may underestimate RE-induced adaptations (Bamman et al., 2001; Nindl et al., 2009). Overall, RE responses are largely governed by volume and fatigue, and even when circulating changes are modest, intramuscular transcript-level adaptations may emerge more clearly, with a delay during the recovery period.

## 4 Exerkines: muscle–brain crosstalk and blood–brain barrier

### 4.1 Exerkine transport to the brain: mechanisms and controversies

Whether exerkines traverse the BBB is central to interpreting the muscle–brain axis. Mechanisms include indirect BBB mediated signaling without translocation and direct transport of selected exerkines into the CNS (Banks et al., 1994; Jones and Shusta, 2007; Kostka et al., 2024; Licinio and Wong, 1997). Even for exerkines proposed to enter the CNS directly, studies disagree on whether BBB transport occurs and, if so, on its magnitude, as well as whether the CNS-accessible form is an intact protein or a fragment (Banks et al., 1994; Pan et al., 1998; Pardridge, 2007). In contrast, IL-6 and IGF-1 have strong radiotracer evidence supporting saturable influx into the brain (Banks et al., 1994; Pan and Kastin, 2000; Threlkeld et al., 2010). For IL-6, the limited recovery of intact ligands in the brain or cerebrospinal fluid (CSF) suggests that the major effects may reflect endothelial IL-6R/STAT3/suppressor of cytokine signaling 3 (SOCS3) signaling or BBB modulation, rather than direct parenchymal action (Blecharz-Lang et al., 2018; Eskilsson et al., 2014). IGF-1 influx is supported by iodine-125-labeled IGF-1 (^125^I–IGF-1), yet IGF-binding proteins (IGFBPs) can alter tracer bioavailability and confound influx estimates, necessitating models that incorporate serum protein transport interactions (Nieto-Estévez et al., 2016; Pan and Kastin, 2000).

FNDC5/irisin and BDNF are often discussed as direct-route candidates; however, their mechanisms remain controversial (Arosio et al., 2021; Kostka et al., 2024; Zhao, 2022). Peripheral irisin elevation following hepatic FNDC5 delivery or repeated recombinant administration is associated with hippocampal BDNF induction, supporting the possibility of brain access (Kim and Leem, 2019; Wrann et al., 2013). However, a specific receptor in the adult brain has not yet been established, and indirect anti-inflammatory, metabolic, or BBB-stabilizing effects may explain its neuroprotective effects (Islam et al., 2021; Kim and Leem, 2019). For BDNF, radiotracer studies report brain entry after peripheral administration, whereas other studies suggest minimal transport or limited passage of intact BDNF, implying dependence on the disease state, BBB integrity, and assay approach (Di Lazzaro et al., 2007; Pan et al., 1998; Wu and Pardridge, 1999). CTSB increases in the brain after intravenous injection, supporting peripheral entry (Moon et al., 2016). However, PE also elevates hippocampal *Ctsb* mRNA levels, indicating substantial local regulation (Moon et al., 2016). Overall, the BBB interactions were best described as graded exerkine-specific uncertainties. Interpreting these functions requires the separation of true CNS influx, molecular integrity, and indirect endothelial contributions (Banks et al., 1994; Jones and Shusta, 2007; Kostka et al., 2024).

### 4.2 Exerkine modulation of blood–brain barrier function in brain disorders

AD, PD, PTSD, and depressive/anxiety disorders are frequently associated with systemic inflammation and, in some patients, metabolic dysfunction (including insulin resistance), which may activate the cerebral endothelium and compromise BBB integrity (Alkhalifa et al., 2023; Lau et al., 2024; Mehdi et al., 2023; Ritson et al., 2024; Sah and Singewald, 2025). Pro-inflammatory cytokines such as tumor necrosis factor-alpha (TNF-α) and IL-1β can increase oxidative stress, activate matrix metalloproteinases (MMPs), destabilize tight junctions, and increase BBB permeability, thereby promoting immune cell infiltration and microglia-driven synaptic dysfunction (Lau et al., 2024; Sweeney et al., 2018). In parallel, insulin resistance reduces insulin delivery to the brain, thereby impairing cerebrovascular function, metabolic homeostasis, and immune regulation (Heni et al., 2014; Rhea et al., 2018; Wang et al., 2022).

BDNF/TrkB signaling attenuates IL-1β- and TNF-α-induced endothelial hyperpermeability and prevents the cytokine-induced reduction in VE-cadherin protein levels (Matsuda et al., 2015). Endothelial TrkB activation can also stabilize vascular endothelial protein tyrosine phosphatase (VE-PTP)/VE-cadherin coupling and limit VE-cadherin cleavage, thereby maintaining junctional organization (Jiang et al., 2015). Consistent with this, VE-cadherin at adherens junctions helps preserve tight-junction integrity by promoting claudin-5 expression and tight-junction organization at the endothelial interface (Taddei et al., 2008). Beyond endothelial junctions, BDNF may reinforce the pericyte–endothelial axis by activating TrkB in mural cells (Anastasia et al., 2014). In addition, BDNF-mediated anti-inflammatory effects on microglia may indirectly favor barrier protection/recovery by reducing inflammatory stress (Charlton et al., 2023).

IGF-1 supports BBB integrity in a context-dependent manner via endothelial IGF-1R signaling (Gulej et al., 2024; Higashi et al., 2020). In ischemic models, IGF-I modulates BBB endothelial function and PI3K signaling (Bake et al., 2019, Bake et al., 2016). IGF-1 also promotes Akt-dependent endothelial nitric oxide synthase (eNOS) phosphorylation and is linked to reduced inflammation and oxidative stress (Higashi et al., 2010; Michell et al., 1999; Sukhanov et al., 2007). However, in neonatal lipopolysaccharide (LPS)-exposed rats, IGF-1 can exacerbate BBB leakage and intracerebral hemorrhage, highlighting age- and inflammatory context-dependent effects (Pang et al., 2010).

Meanwhile, exerkines such as IL-6 and CTSB display context-dependent actions on BBB integrity (Menard et al., 2017; Pedersen and Febbraio, 2008; Rose-John, 2012). IL-6 trans-signaling is pro-inflammatory and can activate endothelial cells (Rose-John, 2012), claudin-5 loss permits peripheral IL-6 brain entry and depression-like behavior (Menard et al., 2017). Conversely, the transient, PE-induced surge of muscle-derived IL-6 stimulates anti-inflammatory mediators and may indirectly support BBB stability by dampening systemic inflammation (Pedersen and Febbraio, 2008; Petersen and Pedersen, 2005). Pathological CTSB activity (e.g., after ischemic injury) may support a possible CTSB/calpain-associated contribution to neurovascular injury with MMP-9 upregulation after ischemia (Tsubokawa et al., 2006).

## 5 Therapeutic potential of exerkines in neurodegenerative and mental disorders

### 5.1 Alzheimer’s disease

AD is characterized by an imbalance in Aβ production, aggregation, and clearance, together with tau hyperphosphorylation and neurofibrillary tangle accumulation (Hampel et al., 2021; Long and Holtzman, 2019). These pathological axes reinforce one another through neuroinflammation and synaptic dysfunction (Hampel et al., 2021; Long and Holtzman, 2019). Accumulating evidence indicates that exerkines modulate glial programs involved in Aβ clearance and synaptic resilience (Kim et al., 2023; Lourenco et al., 2019). Through astrocytes and microglia, exerkines regulate Aβ-degrading enzymes, phagocytic clearance, and broader cytokine/chemokine signaling (Fakhoury, 2018; Kim et al., 2023). Irisin is reduced in the AD hippocampus and CSF, and restoring FNDC5/irisin improves synaptic plasticity and memory in AD models (Lourenco et al., 2019). Mechanistically, irisin binds astrocytic integrin αVβ5, downregulates extracellular signal-regulated kinase (ERK)/STAT3 signaling, and increases extracellular neprilysin release, thereby enhancing Aβ clearance (Kim et al., 2023). In humans, CSF irisin correlates positively with Aβ42, BDNF, and mini-mental state examination (MMSE) scores. This finding may be related to a muscle–brain endocrine link (Lourenco et al., 2020).

BDNF is a key neurotrophin implicated in synaptic function/plasticity in Alzheimer’s models and in PE-related neuroprotection (Belaya et al., 2020; Lourenco et al., 2019; Pena et al., 2020). In male 5xFAD mice, voluntary wheel running improves learning and memory and restores astrocyte-associated hippocampal BDNF signaling, suggesting trophic reprogramming may improve network output (Belaya et al., 2020). Recent research emphasizes neurotrophin receptor signaling, and the p75NTR modulator LM11A-31 showed safety with exploratory biomarker/imaging signals in mild-to-moderate AD (Shanks et al., 2024). CTSB appears context- and stage-dependent. It may worsen Aβ proteotoxicity under sustained burden, yet it may also promote microglial Aβ phagocytosis via PI3K/Akt signaling (Hook et al., 2020; Jiang et al., 2025; Siddiqui et al., 2024). Thus, CTSB activity, localization, and disease stage remain key interpretive variables. In female 3xTg-AD mice, treadmill exercise increased hippocampal IGF-1, whereas hippocampal BDNF and skeletal muscle mature cathepsin B (CTSB/*Ctsb*) were not significantly altered (Pena et al., 2020). RE reduced hippocampal Aβ without significant changes in hippocampal BDNF or skeletal muscle *Ctsb*, suggesting Aβ modulation can occur independent of CTSB/BDNF upregulation and may be context dependent (Pena et al., 2020). IL-6 effects depend on signaling mode and compartment, so transient PE-induced IL-6 responses may differ from chronic IL-6 activation in AD (Pedersen and Febbraio, 2008; Rose-John, 2012). In male 3xTg-AD mice, resistance ladder training increased hippocampal and frontal cortical *IL-6* mRNA without changing serum IL-6, alongside reduced neuroinflammation, improved cognition, and lower Aβ and tau pathology (Liu et al., 2020). By contrast, persistent IL-6 pathway activation in AD-related contexts may be detrimental, as IL-6 deficiency suppresses STAT3-associated cGAS-STING signaling and reduces neuroinflammation and Aβ pathology (Liu et al., 2024). Collectively, these findings suggest transient PE-like activation may fundamentally diverge from chronic pathway engagement.

In this context, IL-6 intersects chemokine networks that govern glial state transitions and immune-cell recruitment. *In vivo* IL-1β challenge amplifies astrocyte-associated C-C motif chemokine ligand 2 (*Ccl2)* and *IL-6* responses, whereas C-X3-C motif chemokine receptor 1 (*Cx3cr1)* deficiency in 5xFAD microglia drives a *Ccl2*-high degenerative program (Lopez-Rodriguez et al., 2021; Puntambekar et al., 2022). In humans, rising circulating monocyte chemoattractant protein-1 (MCP-1; also known as CCL2) tracks poorer memory and, in genotype-stratified analyses, higher AD risk and neuropathology (Bettcher et al., 2019; Huang et al., 2025). High endurance-training status is associated with lower age-related plasma MCP-1, and PE normalizes elevated serum and brain MCP-1 in 3xTg-AD mice (Balan et al., 2021; Haskins et al., 2016). Acute endurance PE increases C-X3-C motif chemokine ligand 1 (CX3CL1), and peripheral CX3CL1 enhances hippocampal BDNF and recognition memory in aged mice (Catoire et al., 2014; Takei et al., 2022). Microglial IGF-1 signaling can enhance astrocytic phagocytosis, whereas long R3 IGF-1 remodels plaques without cognitive rescue in 5xFAD mice (Engel et al., 2025; Zhang et al., 2026). Collectively, these findings support stage- and mechanism-tailored combination strategies that integrate amyloid clearance, inflammatory control, and synaptic resilience.

### 5.2 Parkinson’s disease

PD is a progressive neurodegenerative disorder marked by degeneration of nigrostriatal dopaminergic neurons and Lewy pathology enriched in misfolded α-synuclein (α-syn) (Leak et al., 2024), producing motor impairment and prominent cognitive and affective symptoms (Alfaidi et al., 2024; Hoglinger et al., 2024). Recent studies on PD and α-syn increasingly converge on interconnected hubs involving transcription factor EB (TFEB)-regulated autophagy–lysosomal function, mitochondrial homeostasis, and glia-driven neuroimmune crosstalk (Giamogante et al., 2024; Ma et al., 2025). These networks also encompass oligodendrocytic prosaposin (PSAP)/G protein-coupled receptor 37 (GPR37)/IL-6 signaling and impaired neurotrophin receptor signaling (Giamogante et al., 2024; Ma et al., 2025). Mechanistically, α-syn can bind the TrkB kinase domain and inhibit BDNF/TrkB signaling by disrupting TrkB trafficking and ubiquitination (Kang et al., 2017). In addition, pS129/α-syn promotes membrane retention of TrkB, collapsing the BDNF/ERK/CREB/mTOR loop and further decreasing BDNF expression (Ma et al., 2025). Within this framework, α-syn overexpression reduces mitochondria-lysosome apposition and perturbs local Ca^2+^ transfer, alters Ca^2+^-dependent TFEB nuclear translocation and lysosomal homeostatic transcription (Giamogante et al., 2024). Lysosomal proteases, particularly CTSB, sit at this convergence point. CTSB inhibition impairs autophagy, increases lysosomal cargo accumulation, reduces glucocerebrosidase (GCase/GBA1) activity, and diminishes clearance of pre-formed α-syn fibrils (Jones-Tabah et al., 2024). Conversely, CTSB activation promotes fibril clearance in cellular models and human iPSC-derived dopaminergic lineages, although some cleavage products may show increased aggregation propensity under specific conditions (Jones-Tabah et al., 2024). Thus, CTSB biology appears beneficial but context dependent.

Against this backdrop, exerkines can function as hub modulators, and FNDC5/irisin is emerging as a multi-node regulator extending beyond mitochondrial protection toward immune-autophagy reprogramming (Cai et al., 2026; Zhang et al., 2023). Irisin enhances autophagic flux and restores TFEB-driven lysosomal function by regulating receptor for advanced glycation end products (RAGE) ubiquitination, while promoting NOD-like receptor family pyrin domain containing 3 (NLRP3) inflammasome degradation to suppress microglial inflammation (Cai et al., 2026). Irisin also attenuates oxidative stress and improves mitochondrial complex I activity through integrin-dependent Akt and ERK1/2 signaling, supporting mitochondrial biogenesis and normalized dynamics (Zhang et al., 2023). These mechanisms align with findings from PE studies in the MPTP model of Parkinson’s disease, a neurotoxin model that damages nigrostriatal dopaminergic neurons. Treadmill training increased irisin/FNDC5 in serum and brain regions, reduced α-syn and neuroinflammation, and improved motor outcomes, tyrosine hydroxylase (TH)-positive neuron preservation, neurogenesis, memory, and dopamine-related indices (Tang et al., 2023; Wang et al., 2025; Zhao et al., 2025). In MPTP models, treadmill exercise consistently enhances FNDC5/irisin signaling and downstream neuroprotection—improving BDNF-linked synaptic/cognitive function (Tang et al., 2023), suppressing microglia-driven inflammation via AMPK/silent mating type information regulation 2 homolog 1 (SIRT1) while preserving TH-positive neurons and motor function (Wang et al., 2025), and reducing α-syn/NLRP3 pathology while restoring neurogenesis and memory (Zhao et al., 2025). Neurotrophic signaling is tightly interwoven with this response: aerobic treadmill exercise increased hippocampal FNDC5 together with BDNF and improved depression-like behavior and synaptic structure (Shan et al., 2025).

In PD, neuroimmune convergence includes oligodendroglial PSAP/GPR37 signaling driving neuroinflammation, while IL-6 effects remain source-, sex-, and context-dependent (Chen et al., 2025; Ma et al., 2025). IL-6, MCP-1, and CX3CL1 support a central-peripheral inflammatory axis (Qu et al., 2023). MCP-1 recruits monocytes, amplifies neuroinflammation (Singh et al., 2021), and predicts memory decline, whereas CX3CL1/CX3CR1 counter-regulates microglia and preserves dopaminergic neurons, although context remains dependent (Pabon et al., 2011; Szymura et al., 2020). CX3CL1 is PE-responsive (Della Gatta et al., 2014; Swalsingh et al., 2025). Finally, IGF-1 associates with PD risk, while IGF1R transcytosis supports α-syn immunotherapy delivery (An et al., 2025; Gao et al., 2025; Shin et al., 2022).

### 5.3 Mental disorders

Depression, anxiety disorders, and PTSD converge on dysfunction within prefrontal–limbic circuitry, particularly the prefrontal cortex–hippocampus–amygdala network, supporting shared circuit-level mechanisms across diagnostic boundaries (Kredlow et al., 2022; Maren and Holmes, 2016; Tozzi et al., 2024). Within this network, maladaptive stress responsivity, including HPA-axis dysregulation, interacts with neuroimmune disruption to undermine synaptic plasticity and compromise emotional learning and updating (Feng et al., 2025; Lawrence and Scofield, 2024; Maren and Holmes, 2016).

Evidence increasingly supports inflammation-linked subtypes across mood and stress-related disorders, motivating biomarker-guided stratification (Miller, 2025; Sah and Singewald, 2025; Zeng et al., 2024), and meta-analytic evidence suggests that immunomodulatory interventions yield larger benefits in high-inflammation subgroups (Giollabhui et al., 2026). Within this subtype-oriented framework, IL-6 and chemokine signaling appear central. Meta-analyses consistently report elevated IL-6 in major depressive disorder, and IL-6 is repeatedly implicated in PTSD-associated inflammatory signatures (Dowlati et al., 2010; Haapakoski et al., 2015; Passos et al., 2015). CCL2 has also emerged as a candidate chemokine altered in depression (Leighton et al., 2018). Mechanistically, IL-6 trans-signaling can induce endothelial CCL2 through JAK/STAT3 and PI3K/Akt pathways, providing a plausible route from peripheral inflammatory tone to vascular/BBB remodeling and central immune-cell mobilization (Zegeye et al., 2018). By contrast, neuron-derived CX3CL1 tunes microglial CX3CR1 signaling and synaptic homeostasis (Sheridan and Murphy, 2013), and a cohort study suggests a functional dissociation in PTSD, with CCL2 linked to onset risk and CX3CL1 to resilience (Zhang et al., 2020).

PE may recalibrate this circuit-immune coupling through exerkines that connect peripheral physiology to central plasticity (Severinsen and Pedersen, 2020). By modulating NF-κB–linked inflammatory signaling and microglial states, PE may shape downstream trophic pathways, including BDNF and IGF-1 (Mee-Inta et al., 2019; Strohm and Majewska, 2024). FNDC5/irisin exemplifies this integration: in a single prolonged stress (SPS) PTSD model, exogenous irisin reduced anxiety-like behavior and rescued fear-extinction deficits through an AMPK-dependent mechanism, increasing p-AMPK in the hippocampus, frontal cortex, and amygdala while suppressing NF-κB-linked inflammatory mediators (Xie et al., 2025). Complementary work further suggests microglial integrin αVβ5/AMPK signaling and autophagy engagement as part of this anti-inflammatory irisin response (Zhang et al., 2024).

PE studies in rodent depression- and PTSD-related stress models reinforce a coupled trophic–inflammatory response to physical training. In chronic stress paradigms, treadmill exercise preconditioning upregulates hippocampal FNDC5/BDNF signaling and improves stress-related behavior (Babaei et al., 2021), increases hippocampal BDNF and IGF-1 alongside synaptic plasticity readouts and behavioral recovery (Kang et al., 2020), and attenuates hippocampal inflammatory cytokines including IL-6 and IL-1β (Xiao et al., 2021). In PTSD paradigms, treadmill exercise improves anxiety-like behavior and is accompanied by increased neurotrophic support (BDNF, and in some designs IGF-1) in stress-relevant regions, with effect magnitude shaped by hormonal status and the timing of training relative to the stressor (Amiri et al., 2024; Eshaghi-Gorji et al., 2024; Mirjalili et al., 2022). Collectively, these findings support a subtype-sensitive cascade in which elevated inflammatory tone may constrain neuroimmune signaling, whereas PE-responsive FNDC5/irisin-linked trophic pathways (BDNF/IGF-1) promote plasticity relevant to affect regulation and stress resilience.

## 6 Conclusion and future perspectives

This review argues that exerkines mediate muscle–brain crosstalk across AD, PD, and stress-related mental disorders. FNDC5/irisin, BDNF, CTSB, IL-6, and IGF-1 show modality-, intensity-, and time-dependent kinetics and may reprogram glial pathways governing proteostasis, mitochondria–lysosome function, inflammatory tone, and synaptic plasticity, potentially *via* BBB trafficking and endothelial signaling. Human studies remain difficult to compare due to heterogeneous biospecimens, inconsistent sampling timepoints and fasting status, variable preprocessing, and limited normalization to PE dose, muscle mass, and fitness. Preclinical work rarely establishes molecule-specific causality because paradigms are nonstandardized, full-length proteins versus cleavage products are not consistently distinguished, BBB protein/EV tracing is incomplete, muscle origin is often unconfirmed, and secretion kinetics are poorly aligned with human datasets. Under characterized candidates such as CX3CL1 and MCP-1 may be relevant to inflammatory subtypes of depression, anxiety, and PTSD, yet their regulation and causal roles remain unclear. Priority next steps include standardized workflows, harmonized longitudinal assays, origin-confirming tagging with protein/EV tracing, and multi-omics discovery with causal validation.

## Glossary

^125^I–IGF-1: iodine-125–labeled insulin-like growth factor-1
5′-UTR: 5′ untranslated region
Aβ: amyloid-β
AD: Alzheimer’s disease
ADAM: a disintegrin and metalloproteinase
AE: aerobic exercise
Akt: protein kinase B (PKB)
ALS: amyotrophic lateral sclerosis
AMPK: AMP-activated protein kinase
α-syn: alpha-synuclein
BBB: blood–brain barrier
BBS: Berg Balance Scale
BDNF: brain-derived neurotrophic factor
CNS: central nervous system
CRP: C-reactive protein
CSF: cerebrospinal fluid
CTSB: cathepsin B
CX3CL1: C-X3-C motif chemokine ligand 1 (fractalkine)
CX3CR1: C-X3-C motif chemokine receptor 1 (fractalkine receptor)
DCX: doublecortin
eNOS: endothelial nitric oxide synthase
EPS: extrapyramidal symptoms
ER: endoplasmic reticulum
ERK: extracellular signal-regulated kinase
EVs: extracellular vesicles
GH: growth hormone
GPR37: G protein-coupled receptor 37 (prosaposin receptor)
HIIT: high-intensity interval training
HPA: hypothalamic–pituitary–adrenal (axis)
IGF-1: insulin-like growth factor-1
IGFBP-3: insulin-like growth factor binding protein-3
IL-1β: interleukin-1 beta
IL-6: interleukin-6
IL-6Rα: interleukin-6 receptor alpha
LPS: lipopolysaccharide
LTP: long-term potentiation
MAPK: mitogen-activated protein kinase
MCP-1: monocyte chemoattractant protein-1 (CCL2)
MDD: major depressive disorder
MGF: mechano growth factor
MMPs: matrix metalloproteinases
mTORC1: mechanistic target of rapamycin complex 1
MPTP: 1-methyl-4-phenyl-1,2,3,6-tetrahydropyridine
NGF: nerve growth factor
NLRP3: NLR family pyrin domain containing 3
PD: Parkinson’s disease
PE: physical exercise
PGC-1α: peroxisome proliferator-activated receptor gamma coactivator 1-alpha
PI3K: phosphoinositide 3-kinase
PSAP: prosaposin
PTSD: post-traumatic stress disorder
RAGE: receptor for advanced glycation end products (AGER)
RE: resistance exercise
sIL-6R: soluble interleukin-6 receptor
SIRT1: sirtuin 1
SOCS3: suppressor of cytokine signaling 3
SPS: single prolonged stress
STAT3: signal transducer and activator of transcription 3
STAT5B: signal transducer and activator of transcription 5B
TFEB: transcription factor EB
TH: tyrosine hydroxylase
TNF-α: tumor necrosis factor-alpha
TrkB: tropomyosin receptor kinase B
UPS: ubiquitin–proteasome system
VE-PTP: vascular endothelial protein tyrosine phosphatase

## References

[B1] Ahuja P. Ng C. F. Pang B. P. S. Chan W. S. Tse M. C. L. Bi X. . (2022). (brain derived neurotrophic factor) maintains mitochondrial quality control in female mice. Autophagy 18, 1367–1384. doi: 10.1080/15548627.2021.1985257, PMID: 34689722 PMC9225428

[B2] Aid T. Kazantseva A. Piirsoo M. Palm K. Timmusk T. (2007). Mouse and rat BDNF gene structure and expression revisited. J. Neurosci. Res. 85, 525–535. doi: 10.1002/jnr.21139, PMID: 17149751 PMC1878509

[B3] Alfaidi M. Barker R. A. Kuan W. L. (2024). An update on immune-based alpha-synuclein trials in Parkinson’s disease. J. Neurol. 272, 21. doi: 10.1007/s00415-024-12770-x, PMID: 39666171 PMC11638298

[B4] Alkhalifa A. E. Al-Ghraiybah N. F. Odum J. Shunnarah J. G. Austin N. Kaddoumi A. (2023). Blood-brain barrier breakdown in alzheimer’s disease: mechanisms and targeted strategies. Int. J. Mol. Sci. 24 (22), 16288 doi: 10.3390/ijms242216288, PMID: 38003477 PMC10671257

[B5] Alzheimer’s Association (2025). 2025 Alzheimer’s disease facts and figures. Alzheimers Dementia 21, e70235. doi: 10.1002/alz.70235, PMID: 41837764

[B6] Amiri A. Janbazi M. Jamali S. R. Ehsani Vostacolaee S. Shafia S. (2024). Impact of physical exercise on behavioral functions and neurotrophic factors on ovariectomized rats exposed to SPS. Res. Mol. Med. 12, 93–106.

[B7] An S. McInnis J. J. Kim D. Li Y. Tasdemir-Yilmaz O. Ahn J. . (2025). A brain-shuttled antibody targeting alpha synuclein aggregates for the treatment of synucleinopathies. NPJ Parkinsons Dis. 11, 254. doi: 10.1038/s41531-025-01117-6, PMID: 40847026 PMC12373799

[B8] Anastasia A. Deinhardt K. Wang S. Martin L. Nichol D. Irmady K. . (2014). Trkb signaling in pericytes is required for cardiac microvessel stabilization. PloS One 9, e87406. doi: 10.1371/journal.pone.0087406, PMID: 24498100 PMC3909185

[B9] Annibalini G. Contarelli S. Lucertini F. Guescini M. Maggio S. Ceccaroli P. . (2019). Muscle and systemic molecular responses to a single flywheel based iso-inertial training session in resistance-trained men. Front. Physiol. 10. doi: 10.3389/fphys.2019.00554, PMID: 31143128 PMC6521220

[B10] Archundia-Herrera C. Macias-Cervantes M. Ruiz-Muñoz B. Vargas-Ortiz K. Kornhauser C. Perez-Vazquez V. (2017). Muscle irisin response to aerobic vs HIIT in overweight female adolescents. Diabetol. Metab. Syndrome 9, 101. 10.1186/s13098-017-0302-5 PMC5746008 29299068

[B11] Arnold P. Luckstadt W. Li W. Boll I. Lokau J. Garbers C. . (2020). Joint reconstituted signaling of the IL-6 receptor via extracellular vesicles. Cells 9 (5), 1307 doi: 10.3390/cells9051307, PMID: 32456348 PMC7291149

[B12] Arosio B. Guerini F. R. Voshaar R. C. O. Aprahamian I. (2021). Blood brain-derived neurotrophic factor (BDNF) and major depression: do we have a translational perspective? Front. Behav. Neurosci. 15. doi: 10.3389/fnbeh.2021.626906, PMID: 33643008 PMC7906965

[B13] Babaei A. Nourshahi M. Fani M. Entezari Z. Jameie S. B. Haghparast A. (2021). The effectiveness of continuous and interval exercise preconditioning against chronic unpredictable stress: Involvement of hippocampal PGC-1alpha/FNDC5/BDNF pathway. J. Psychiatr. Res. 136, 173–183. doi: 10.1016/j.jpsychires.2021.02.006, PMID: 33607579

[B14] Bai H. Yang B. Yu W. Xiao Y. Yu D. Zhang Q. (2018). Cathepsin B links oxidative stress to the activation of NLRP3 inflammasome. Exp. Cell Res. 362, 180–187. doi: 10.1016/j.yexcr.2017.11.015, PMID: 29196167

[B15] Bake S. Okoreeh A. K. Alaniz R. C. Sohrabji F. (2016). Insulin-like growth factor (IGF)-I modulates endothelial blood-brain barrier function in ischemic middle-aged female rats. Endocrinology 157, 61–69. doi: 10.1210/en.2015-1840, PMID: 26556536 PMC4701884

[B16] Bake S. Okoreeh A. Khosravian H. Sohrabji F. (2019). Insulin-like Growth Factor (IGF)-1 treatment stabilizes the microvascular cytoskeleton under ischemic conditions. Exp. Neurol. 311, 162–172. doi: 10.1016/j.expneurol.2018.09.016, PMID: 30287160 PMC6263796

[B17] Balan E. Diman A. Everard A. Nielens H. Decottignies A. Deldicque L. (2021). Endurance training alleviates MCP-1 and TERRA accumulation at old age in human skeletal muscle. Exp. Gerontology 153, 111510. doi: 10.1016/j.exger.2021.111510, PMID: 34371098

[B18] Bamman M. M. Shipp J. R. Jiang J. Gower B. A. Hunter G. R. Goodman A. . (2001). Mechanical load increases muscle IGF-I and androgen receptor mRNA concentrations in humans. Am. J. Physiol. Endocrinol. Metab. 280, E383–E390. doi: 10.1152/ajpendo.2001.280.3.E383, PMID: 11171591

[B19] Banks W. A. Kastin A. J. Gutierrez E. G. (1994). Penetration of interleukin-6 across the murine blood-brain barrier. Neurosci. Lett. 179, 53–56. doi: 10.1016/0304-3940(94)90933-4, PMID: 7845624

[B20] Barde Y. A. (2025). The physiopathology of brain-derived neurotrophic factor. Physiol. Rev. 105, 2073–2140. doi: 10.1152/physrev.00038.2024, PMID: 40490314

[B21] Bawa P. N. Jones K. E. Stein R. B. (2014). Assessment of size ordered recruitment. Front. Hum. Neurosci. 8. doi: 10.3389/fnhum.2014.00532, PMID: 25120446 PMC4112781

[B22] Baxter R. C. (2024). Endocrine and cellular physiology and pathology of the insulin-like growth factor acid-labile subunit. Nat. Rev. Endocrinol. 20, 414–425. doi: 10.1038/s41574-024-00970-4, PMID: 38514815

[B23] Belaya I. Ivanova M. Sorvari A. Ilicic M. Loppi S. Koivisto H. . (2020). Astrocyte remodeling in the beneficial effects of long-term voluntary exercise in Alzheimer’s disease. J. Neuroinflamm. 17, 271. 10.1186/s12974-020-01935-w PMC7493971 32933545

[B24] Bettariga F. Taaffe D. R. Galvao D. A. Lopez P. Bishop C. Markarian A. M. . (2024). Exercise training mode effects on myokine expression in healthy adults: A systematic review with meta-analysis. J. Sport Health Sci. 13, 764–779. doi: 10.1016/j.jshs.2024.04.005, PMID: 38604409 PMC11336361

[B25] Bettcher B. M. Neuhaus J. Wynn M. J. Elahi F. M. Casaletto K. B. Saloner R. . (2019). Increases in a pro-inflammatory chemokine, MCP-1, are related to decreases in memory over time. Front. Aging Neurosci. 11. doi: 10.3389/fnagi.2019.00025, PMID: 30814948 PMC6381047

[B26] Blecharz-Lang K. G. Wagner J. Fries A. Nieminen-Kelhä M. Rösner J. Schneider U. C. . (2018). Interleukin 6-mediated endothelial barrier disturbances can be attenuated by blockade of the IL6 receptor expressed in brain microvascular endothelial cells. Trans. Stroke Res. 9, 631–642. doi: 10.1007/s12975-018-0614-2, PMID: 29429002

[B27] Bloem B. R. Okun M. S. Klein C. (2021). Parkinson’s disease. Lancet 397, 2284–2303. 33848468 10.1016/S0140-6736(21)00218-X

[B28] Bonetto A. Aydogdu T. Jin X. Zhang Z. Zhan R. Puzis L. . (2012). JAK/STAT3 pathway inhibition blocks skeletal muscle wasting downstream of IL-6 and in experimental cancer cachexia. Am. J. Physiol. Endocrinol. Metab. 303, E410–E421. doi: 10.1152/ajpendo.00039.2012, PMID: 22669242 PMC3423125

[B29] Boström W. Jedrychowski M. P. Korde A. Ye L. Lo J. C. Rasbach K. A. . (2012). A PGC1-α-dependent myokine that drives brown-fat-like development of white fat and thermogenesis. Nature 481, 463–468. doi: 10.1038/nature10777, PMID: 22237023 PMC3522098

[B30] Brix K. . (2018). Host cell proteases: Cathepsins. In: Activation of viruses by host proteases. Cham: Springer International Publishing. 249–276. doi: 10.1007/978-3-319-75474-1_10, PMID:

[B31] Cai L. Liu Y. Tang S. Deng S. Zhang L. Wang Y. . (2026). Targeting microglial inflammation in Parkinson’s disease: irisin activates PAFAH1B1-RAGE ubiquitination and TFEB-dependent autophagy to alleviate neurodegeneration. Commun. Biol. 9, 114. doi: 10.1038/s42003-025-09389-7, PMID: 41520051 PMC12848009

[B32] Cámara-Calmaestra R. Martínez-Amat A. Aibar-Almazán A. Hita-Contreras F. de Miguel Hernando N. Achalandabaso-Ochoa A. (2022). Effectiveness of physical exercise on alzheimer’s disease. a systematic review. J. Prev. Alzheimer’s Dis. 9, 601–616. 36281664 10.14283/jpad.2022.57

[B33] Campos H. C. Ribeiro D. E. Hashiguchi D. Glaser T. Milanis M. d. S. . (2023). Neuroprotective effects of resistance physical exercise on the APP/PS1 mouse model of Alzheimer’s disease. Front. Neurosci. 17, 1132825. 37090809 10.3389/fnins.2023.1132825 PMC10116002

[B34] Carro E. Trejo J. L. Gomez-Isla T. LeRoith D. Torres-Aleman I. (2002). Serum insulin-like growth factor I regulates brain amyloid-beta levels. Nat. Med. 8, 1390–1397. doi: 10.1038/nm1202-793, PMID: 12415260

[B35] Casarotto P. Umemori J. Castren E. (2022). BDNF receptor TrkB as the mediator of the antidepressant drug action. Front. Mol. Neurosci. 15. doi: 10.3389/fnmol.2022.1032224, PMID: 36407765 PMC9666396

[B36] Catoire M. Mensink M. Kalkhoven E. Schrauwen P. Kersten S. (2014). Identification of human exercise-induced myokines using secretome analysis. Physiol. Genomics 46, 256–267. doi: 10.1152/physiolgenomics.00174.2013, PMID: 24520153

[B37] Charlton T. Prowse N. McFee A. Heiratifar N. Fortin T. Paquette C. . (2023). Brain-derived neurotrophic factor (BDNF) has direct anti-inflammatory effects on microglia. Front. Cell. Neurosci. 17. doi: 10.3389/fncel.2023.1188672, PMID: 37404293 PMC10315457

[B38] Chen F. Duan Y. Wang M. Liu Z. Zhao J. Fan G. . (2025). Sex-dependent impact of Il6 deficiency in Parkinson’s disease mice. Genes Dis., 101986. doi: 10.1016/j.gendis.2025.101986, PMID: 41836151

[B39] Chi C. Fu H. Li Y. H. Zhang G. Y. Zeng F. Y. Ji Q. X. . (2022). Exerkine fibronectin type-III domain-containing protein 5/irisin-enriched extracellular vesicles delay vascular ageing by increasing SIRT6 stability. Eur. Heart J. 43, 4579–4595. doi: 10.1093/eurheartj/ehac431, PMID: 35929617

[B40] Cho H. C. Kim J. Kim S. Son Y. H. Lee N. Jung S. H. (2012). The concentrations of serum, plasma and platelet BDNF are all increased by treadmill VO(2)max performance in healthy college men. Neurosci. Lett. 519, 78–83. doi: 10.1016/j.neulet.2012.05.025, PMID: 22617010

[B41] Chow L. S. Gerszten R. E. Taylor J. M. Pedersen B. K. van Praag H. Trappe S. . (2022). Exerkines in health, resilience and disease. Nat. Rev. Endocrinol. 18, 273–289. doi: 10.1038/s41574-022-00641-2, PMID: 35304603 PMC9554896

[B42] Clow C. Jasmin B. J. (2010). Brain-derived neurotrophic factor regulates satellite cell differentiation and skeltal muscle regeneration. Mol. Biol. Cell 21, 2182–2190. doi: 10.1091/mbc.E10-02-0154, PMID: 20427568 PMC2893983

[B43] Colaianni G. Cuscito C. Mongelli T. Pignataro P. Buccoliero C. Liu P. . (2015). The myokine irisin increases cortical bone mass. Proc. Natl. Acad. Sci. U.S.A. 112, 12157–12162. doi: 10.1073/pnas.1516622112, PMID: 26374841 PMC4593131

[B44] Collaborators C.-M. D. (2021). Global prevalence and burden of depressive and anxiety disorders in 204 countries and territories in 2020 due to the COVID-19 pandemic. Lancet 398, 1700–1712. doi: 10.1016/S0140-6736(21)02143-7, PMID: 34634250 PMC8500697

[B45] Collaborators G. B. D. N. S. D. (2024). Global, regional, and national burden of disorders affecting the nervous system 1990-2021: a systematic analysis for the Global Burden of Disease Study 2021. Lancet Neurol. 23, 344–381. doi: 10.1016/S1474-4422(24)00038-3, PMID: 38493795 PMC10949203

[B46] Copeland J. L. Verzosa M. L. (2014). Endocrine response to an ultra-marathon in pre- and post-menopausal women. Biol. Sport 31, 125–131. doi: 10.5604/20831862.1097480, PMID: 24899777 PMC4042659

[B47] Correia P. R. Pansani A. MaChado F. Andrade M. Silva A. C. Scorza F. A. . (2010). Acute strength exercise and the involvement of small or large muscle mass on plasma brain-derived neurotrophic factor levels. Clinics (Sao Paulo) 65, 1123–1126. doi: 10.1590/s1807-59322010001100012, PMID: 21243284 PMC2999707

[B48] Cui S. F. Li W. Niu J. Zhang C. Y. Chen X. Ma J. Z. (2015). Acute responses of circulating microRNAs to low-volume sprint interval cycling. Front. Physiol. 6. doi: 10.3389/fphys.2015.00311, PMID: 26578983 PMC4626635

[B49] da Cunha L. L. Feter N. Alt R. Rombaldi A. J. (2023). Effects of exercise training on inflammatory, neurotrophic and immunological markers and neurotransmitters in people with depression: A systematic review and meta-analysis. J. Affect. Disord. 326, 73–82. doi: 10.1016/j.jad.2023.01.086, PMID: 36709828

[B50] da Rocha J. F. Lance M. L. Luo R. H. Schlachter P. Moreira L. Iqbal M. A. . (2025). Protective exercise responses in the dentate gyrus of Alzheimer’s disease mouse model revealed with single-nucleus RNA-sequencing. Nat. Neurosci. 28 (7), 154 doi: 10.1038/s41593-025-01971-w, PMID: 40506544 PMC12704105

[B51] De la Rosa A. Solana E. Corpas R. Bartrés-Faz D. Pallàs M. Vina J. . (2019). Long-term exercise training improves memory in middle-aged men and modulates peripheral levels of BDNF and Cathepsin B. Sci. Rep. 9, 3337. doi: 10.1038/s41598-019-40040-8, PMID: 30833610 PMC6399244

[B52] Della Gatta P. A. Cameron-Smith D. Peake J. M. (2014). Acute resistance exercise increases the expression of chemotactic factors within skeletal muscle. Eur. J. Appl. Physiol. 114, 2157–2167. doi: 10.1007/s00421-014-2936-4, PMID: 24968868

[B53] Di Lazzaro V. Profice P. Pilato F. Dileone M. Florio L. Tonali P. A. . (2007). BDNF plasma levels in acute stroke. Neurosci. Lett. 422, 128–130. doi: 10.1016/j.neulet.2007.06.001, PMID: 17590513

[B54] Dowlati Y. Herrmann N. Swardfager W. Liu H. Sham L. Reim E. K. . (2010). A meta-analysis of cytokines in major depression. Biol. Psychiatry 67, 446–457. doi: 10.1016/j.biopsych.2009.09.033, PMID: 20015486

[B55] Duman R. S. Monteggia L. M. (2006). A neurotrophic model for stress-related mood disorders. Biol. Psychiatry 59, 1116–1127. doi: 10.1016/j.biopsych.2006.02.013, PMID: 16631126

[B56] Ellingsgaard H. Seelig E. Timper K. Coslovsky M. Soederlund L. Lyngbaek M. P. . (2020). GLP-1 secretion is regulated by IL-6 signalling: a randomised, placebo-controlled study. Diabetologia 63, 362–373. doi: 10.1007/s00125-019-05045-y, PMID: 31796986

[B57] Engel M. G. Narayan S. Cui M. H. Branch C. A. Zhang X. Gandy S. E. . (2025). Intranasal long R3 insulin-like growth factor-1 treatment promotes amyloid plaque remodeling in cerebral cortex but fails to preserve cognitive function in male 5XFAD mice. J. Alzheimers Dis. 103, 113–126. doi: 10.1177/13872877241299056, PMID: 39610283 PMC12617435

[B58] Erta M. Quintana A. Hidalgo J. (2012). Interleukin-6, a major cytokine in the central nervous system. Int. J. Biol. Sci. 8, 1254–1266. doi: 10.7150/ijbs.4679, PMID: 23136554 PMC3491449

[B59] Eshaghi-Gorji R. Talebpour Amiri F. Mirzae M. Shafia S. Akhoundzadeh K. (2024). Effects of the combination of bone marrow stromal cells and exercise on corticosterone, BDNF, IGF-1, and anxiety-like behaviour in a rat model of post-traumatic stress disorder: Comparable effects of exercise. World J. Biol. Psychiatry 25, 370–383. doi: 10.1080/15622975.2024.2382693, PMID: 39049204

[B60] Eskilsson A. Mirrasekhian E. Dufour S. Schwaninger M. Engblom D. Blomqvist A. (2014). Immune-induced fever is mediated by IL-6 receptors on brain endothelial cells coupled to STAT3-dependent induction of brain endothelial prostaglandin synthesis. J. Neurosci. 34, 15957–15961. doi: 10.1523/Jneurosci.3520-14.2014, PMID: 25429137 PMC6608482

[B61] Fakhoury M. (2018). Microglia and astrocytes in alzheimer’s disease: implications for therapy. Curr. Neuropharmacol 16, 508–518. doi: 10.2174/1570159X15666170720095240, PMID: 28730967 PMC5997862

[B62] Febbraio M. A. Pedersen B. K. (2005). Contraction-induced myokine production and release: is skeletal muscle an endocrine organ? Exercise Sport Sci. Rev. 33, 114–119. doi: 10.1097/00003677-200507000-00003, PMID: 16006818

[B63] Feng X. Jia M. Cai M. Zhu T. Hashimoto K. Yang J. J. (2025). Correction: Central-peripheral neuroimmune dynamics in psychological stress and depression: insights from current research. Mol. Psychiatry 30, 5613. doi: 10.1038/s41380-025-03121-x, PMID: 40954281 PMC12532572

[B64] Fernandez A. M. Torres-Aleman I. (2012). The many faces of insulin-like peptide signalling in the brain. Nat. Rev. Neurosci. 13, 225–239. doi: 10.1038/nrn3209, PMID: 22430016

[B65] Fischer C. P. Plomgaard P. Hansen A. K. Pilegaard H. Saltin B. Pedersen B. K. (2004). Endurance training reduces the contraction-induced interleukin-6 mRNA expression in human skeletal muscle. Am. J. Physiology-Endocrinology Metab. 287, E1189–E1194. doi: 10.1152/ajpendo.00206.2004, PMID: 15304377

[B66] Fukada S. I. Nakamura A. (2021). Exercise/resistance training and muscle stem cells. Endocrinol. Metab. (Seoul) 36, 737–744. doi: 10.3803/EnM.2021.401, PMID: 34372625 PMC8419599

[B67] Gaitan J. M. Moon H. Y. Stremlau M. Dubal D. B. Cook D. B. Okonkwo O. C. . (2021). Effects of aerobic exercise training on systemic biomarkers and cognition in late middle-aged adults at risk for Alzheimer’s disease. Front. Endocrinol. 12. doi: 10.3389/fendo.2021.660181, PMID: 34093436 PMC8173166

[B68] Gao S. Wang Z. Huang Y. Yang G. Wang Y. Yi Y. . (2025). Early detection of Parkinson’s disease through multiplex blood and urine biomarkers prior to clinical diagnosis. NPJ Parkinsons Dis. 11, 35. doi: 10.1038/s41531-025-00888-2, PMID: 39994191 PMC11850829

[B69] Giamogante F. Barazzuol L. Maiorca F. Poggio E. Esposito A. Masato A. . (2024). A SPLICS reporter reveals [Formula: see text]-synuclein regulation of lysosome-mitochondria contacts which affects TFEB nuclear translocation. Nat. Commun. 15, 1516. doi: 10.1038/s41467-024-46007-2, PMID: 38374070 PMC10876553

[B70] Giollabhui N. Madison A. A. Lydston M. Lenoel Quang E. Miller A. H. Liu R. T. (2026). Effect of anti-inflammatory treatment on depressive symptom severity and anhedonia in depressed individuals with elevated inflammation: systematic review and meta-analysis of randomized controlled trials. Am. J. Psychiatry 183, 70–79. doi: 10.1176/appi.ajp.20241115, PMID: 41366844 PMC12910469

[B71] Gogos J. A. Thompson R. Lowry W. Sloane B. F. Weintraub H. Horwitz M. (1996). Gene trapping in differentiating cell lines: Regulation of the lysosomal protease cathepsin B in skeletal myoblast growth and fusion. J. Cell Biol. 134, 837–847. doi: 10.1083/jcb.134.4.837, PMID: 8769410 PMC2120969

[B72] Goodwin V. A. Richards S. H. Taylor R. S. Taylor A. H. Campbell J. L. (2008). The effectiveness of exercise interventions for people with Parkinson’s disease: A systematic review and meta-analysis. Movement Disord. 23, 631–640. 18181210 10.1002/mds.21922

[B73] Greenberg M. E. Xu B. Lu B. Hempstead B. L. (2009). New insights in the biology of BDNF synthesis and release: implications in CNS function. J. Neurosci. 29, 12764–12767. doi: 10.1523/JNEUROSCI.3566-09.2009, PMID: 19828787 PMC3091387

[B74] Gruol D. L. (2015). IL-6 regulation of synaptic function in the CNS. Neuropharmacology 96, 42–54. doi: 10.1016/j.neuropharm.2014.10.023, PMID: 25445486 PMC4446251

[B75] Gulej R. Csik B. Faakye J. Tarantini S. Shanmugarama S. Chandragiri S. S. . (2024). Endothelial deficiency of insulin-like growth factor-1 receptor leads to blood-brain barrier disruption and accelerated endothelial senescence in mice, mimicking aspects of the brain aging phenotype. Microcirculation 31, e12840. doi: 10.1111/micc.12840, PMID: 38082450 PMC10922445

[B76] Guo Q. Luo Q. Song G. (2024). Control of muscle satellite cell function by specific exercise-induced cytokines and their applications in muscle maintenance. J. Cachexia Sarcopenia Muscle 15, 466–476. doi: 10.1002/jcsm.13440, PMID: 38375571 PMC10995279

[B77] Guo L. Yang X. Zhang Y. Xu X. Li Y. (2022). Effect of exercise on cognitive function and synaptic plasticity in Alzheimer’s disease models: A systematic review and meta-analysis. Front. Aging Neurosci. 14. doi: 10.3389/fnagi.2022.1077732, PMID: 36704501 PMC9872519

[B78] Haapakoski R. Mathieu J. Ebmeier K. P. Alenius H. Kivimaki M. (2015). Cumulative meta-analysis of interleukins 6 and 1beta, tumour necrosis factor alpha and C-reactive protein in patients with major depressive disorder. Brain Behav. Immun. 49, 206–215. doi: 10.1016/j.bbi.2015.06.001, PMID: 26065825 PMC4566946

[B79] Hampel H. Hardy J. Blennow K. Chen C. Perry G. Kim S. H. . (2021). The amyloid-beta pathway in alzheimer’s disease. Mol. Psychiatry 26, 5481–5503. doi: 10.1038/s41380-021-01249-0, PMID: 34456336 PMC8758495

[B80] Hashida R. Matsuse H. Kawaguchi T. Yoshio S. Bekki M. Iwanaga S. . (2021). Effects of a low-intensity resistance exercise program on serum miR-630, miR-5703, and fractalkine/CX3CL1 expressions in subjects with no exercise habits: A preliminary study. Hepatol. Res. 51, 823–833. doi: 10.1111/hepr.13670, PMID: 34014020

[B81] Haskins M. Jones T. E. Lu Q. Bareiss S. K. (2016). Early alterations in blood and brain RANTES and MCP-1 expression and the effect of exercise frequency in the 3xTg-AD mouse model of Alzheimer’s disease. Neurosci. Lett. 610, 165–170. doi: 10.1016/j.neulet.2015.11.002, PMID: 26547034

[B82] Heni M. Wagner R. Kullmann S. Veit R. Mat Husin H. Linder K. . (2014). Central insulin administration improves whole-body insulin sensitivity via hypothalamus and parasympathetic outputs in men. Diabetes 63, 4083–4088. doi: 10.2337/db14-0477, PMID: 25028522

[B83] Heyman E. Gamelin F. X. Goekint M. Piscitelli F. Roelands B. Leclair E. . (2012). Intense exercise increases circulating endocannabinoid and BDNF levels in humans--possible implications for reward and depression. Psychoneuroendocrinology 37, 844–851. doi: 10.1016/j.psyneuen.2011.09.017, PMID: 22029953

[B84] Higashi Y. Sukhanov S. Anwar A. Shai S. Y. Delafontaine P. (2010). IGF-1, oxidative stress and atheroprotection. Trends Endocrinol. Metab. 21, 245–254. doi: 10.1016/j.tem.2009.12.005, PMID: 20071192 PMC2848911

[B85] Higashi Y. Sukhanov S. Shai S. Y. Danchuk S. Snarski P. Li Z. . (2020). Endothelial deficiency of insulin-like growth factor-1 receptor reduces endothelial barrier function and promotes atherosclerosis in Apoe-deficient mice. Am. J. Physiol. Heart Circ. Physiol. 319, H730–H743. doi: 10.1152/ajpheart.00064.2020, PMID: 32795184 PMC7654661

[B86] Hoglinger G. U. Adler C. H. Berg D. Klein C. Outeiro T. F. Poewe W. . (2024). A biological classification of Parkinson’s disease: the SynNeurGe research diagnostic criteria. Lancet Neurol. 23, 191–204. doi: 10.1016/S1474-4422(23)00404-0, PMID: 38267191

[B87] Hook V. Yoon M. Mosier C. Ito G. Podvin S. Head B. P. . (2020). Cathepsin B in neurodegeneration of Alzheimer’s disease, traumatic brain injury, and related brain disorders. Biochim. Biophys. Acta Proteins Proteom 1868, 140428. doi: 10.1016/j.bbapap.2020.140428, PMID: 32305689 PMC7261628

[B88] Huang J. Wang Y. Stein T. D. Ang T. F. A. Zhu Y. Tao Q. . (2025). The impact of blood MCP-1 levels on Alzheimer’s disease with genetic variation at the NAV3 and UNC5C loci. Transl. Psychiatry 15, 296. doi: 10.1038/s41398-025-03542-w, PMID: 40830334 PMC12365253

[B89] Hughes D. C. Ellefsen S. Baar K. (2018). Adaptations to endurance and strength training. Cold Spring Harb. Perspect. Med. 8 (6), a029769 doi: 10.1101/cshperspect.a029769, PMID: 28490537 PMC5983157

[B90] Islam M. R. Valaris S. Young M. F. Haley E. B. Luo R. Bond S. F. . (2021). Exercise hormone irisin is a critical regulator of cognitive function. Nat. Metab. 3, 1058–1070. doi: 10.1038/s42255-021-00438-z, PMID: 34417591 PMC10317538

[B91] Jane D. T. Morvay L. Dasilva L. Cavallo-Medved D. Sloane B. F. Dufresne M. J. (2006). Cathepsin B localizes to plasma membrane caveolae of differentiating myoblasts and is secreted in an active form at physiological pH. Biol. Chem. 387, 223–234. doi: 10.1515/BC.2006.030, PMID: 16497156

[B92] Jiang H. Huang S. Li X. Li X. Huang S. Zhang Y. . (2015). Endothelial tyrosine kinase receptor B prevents VE-cadherin cleavage and protects against atherosclerotic lesion development in ApoE-/- mice. Oncotarget 6, 30640–30649. doi: 10.18632/oncotarget.5855, PMID: 26431274 PMC4741558

[B93] Jiang M. Zhao D. Zhou Y. Kong W. Xie Z. Xiong Y. . (2025). Cathepsin B modulates microglial migration and phagocytosis of amyloid beta in Alzheimer’s disease through PI3K-Akt signaling. Neuropsychopharmacology 50, 640–650. doi: 10.1038/s41386-024-01994-0, PMID: 39304744 PMC11845476

[B94] Johnson T. K. Belcher D. J. Sousa C. A. Carzoli J. P. Visavadiya N. P. Khamoui A. V. . (2020). Low-volume acute multi-joint resistance exercise elicits a circulating brain-derived neurotrophic factor response but not a cathepsin B response in well-trained men. Appl. Physiology Nutrition Metab. 45, 1332–1338. 10.1139/apnm-2019-0854 32531180

[B95] Johnson A. L. Kamal M. Parise G. (2023). The role of supporting cell populations in satellite cell mediated muscle repair. Cells 12 (15), 1968 doi: 10.3390/cells12151968, PMID: 37566047 PMC10417507

[B96] Jones A. R. Shusta E. V. (2007). Blood-brain barrier transport of therapeutics via receptor-mediation. Pharm. Res. 24, 1759–1771. doi: 10.1007/s11095-007-9379-0, PMID: 17619996 PMC2685177

[B97] Jones-Tabah J. He K. Karpilovsky N. Senkevich K. Deyab G. Pietrantonio I. . (2024). Correction: The Parkinson’s disease risk gene cathepsin B promotes fibrillar alpha-synuclein clearance, lysosomal function and glucocerebrosidase activity in dopaminergic neurons. Mol. Neurodegener. 19, 94. doi: 10.1186/s13024-024-00791-z, PMID: 39696367 PMC11657430

[B98] Kang J. Wang Y. H. Wang D. (2020). Endurance and resistance training mitigate the negative consequences of depression on synaptic plasticity through different molecular mechanisms. Int. J. Neurosci. 130, 541–550. doi: 10.1080/00207454.2019.1679809, PMID: 31847639

[B99] Kang S. S. Zhang Z. Liu X. Manfredsson F. P. Benskey M. J. Cao X. . (2017). TrkB neurotrophic activities are blocked by alpha-synuclein, triggering dopaminergic cell death in Parkinson’s disease. Proc. Natl. Acad. Sci. U.S.A. 114, 10773–10778. doi: 10.1073/pnas.1713969114, PMID: 28923922 PMC5635931

[B100] Keller C. Steensberg A. Pilegaard H. Osada T. Saltin B. Pedersen B. K. . (2001). Transcriptional activation of the IL-6 gene in human contracting skeletal muscle: influence of muscle glycogen content. FASEB J. 15, 2748–2750. doi: 10.1096/fj.01-0507fje, PMID: 11687509

[B101] Kim E. Kim H. Jedrychowski M. P. Bakiasi G. Park J. Kruskop J. . (2023). Irisin reduces amyloid-beta by inducing the release of neprilysin from astrocytes following downregulation of ERK-STAT3 signaling. Neuron 111, 3619–3633 e3618. doi: 10.1016/j.neuron.2023.08.012, PMID: 37689059 PMC10840702

[B102] Kim M. H. Leem Y. H. (2019). The effects of peripherally-subacute treatment with irisin on hippocampal dendritogenesis and astrocyte-secreted factors. J. Exerc Nutr. Biochem. 23, 32–35. doi: 10.20463/jenb.2019.0029, PMID: 32018344 PMC7004566

[B103] Kim H. J. So B. Choi M. Kang D. Song W. (2015). Resistance exercise training increases the expression of irisin concomitant with improvement of muscle function in aging mice and humans. Exp. Gerontology 70, 11–17. doi: 10.1016/j.exger.2015.07.006, PMID: 26183690

[B104] Kim H. Wrann C. D. Jedrychowski M. Vidoni S. Kitase Y. Nagano K. . (2018). Irisin Mediates Effects on Bone and Fat via alphaV Integrin Receptors. Cell 175(7) 1756–1768, e1717. doi: 10.1016/j.cell.2018.10.025, PMID: 30550785 PMC6298040

[B105] Kostka M. Morys J. Malecki A. Nowacka-Chmielewska M. (2024). Muscle-brain crosstalk mediated by exercise-induced myokines - insights from experimental studies. Front. Physiol. 15. doi: 10.3389/fphys.2024.1488375, PMID: 39687518 PMC11647023

[B106] Kraemer W. J. Ratamess N. A. (2005). Hormonal responses and adaptations to resistance exercise and training. Sports Med. 35, 339–361. doi: 10.2165/00007256-200535040-00004, PMID: 15831061

[B107] Kredlow M. Fenster R. J. Laurent E. S. Ressler K. J. Phelps E. A. (2022). Prefrontal cortex, amygdala, and threat processing: implications for PTSD. Neuropsychopharmacology 47, 247–259. doi: 10.1038/s41386-021-01155-7, PMID: 34545196 PMC8617299

[B108] Lau K. Kotzur R. Richter F. (2024). Blood-brain barrier alterations and their impact on Parkinson’s disease pathogenesis and therapy. Transl. Neurodegener. 13, 37. doi: 10.1186/s40035-024-00430-z, PMID: 39075566 PMC11285262

[B109] Lawrence S. Scofield R. H. (2024). Post traumatic stress disorder associated hypothalamic-pituitary-adrenal axis dysregulation and physical illness. Brain Behav. Immun. Health 41, 100849. doi: 10.1016/j.bbih.2024.100849, PMID: 39280087 PMC11401111

[B110] Leak R. K. Clark R. N. Abbas M. Xu F. Brodsky J. L. Chen J. . (2024). Current insights and assumptions on alpha-synuclein in Lewy body disease. Acta Neuropathologica 148, 18. doi: 10.1007/s00401-024-02781-3, PMID: 39141121 PMC11324801

[B111] Lee T.-H. Ko J.-M. (2025). The effects of 8 weeks of moderate-intensity aerobic exercise on serum irisin and leptin levels in obese women in their 20s. Exercise Sci. 34, 71–80. doi: 10.15857/ksep.2024.00626

[B112] Lee H. J. Lee J. O. Kim N. Kim J. K. Kim H. I. Lee Y. W. . (2015). Irisin, a novel myokine, regulates glucose uptake in skeletal muscle cells via AMPK. Mol. Endocrinol. 29, 873–881. doi: 10.1210/me.2014-1353, PMID: 25826445 PMC5414737

[B113] Lee J. Park J. Kim Y. H. Lee N. H. Song K. M. (2019). Irisin promotes C2C12 myoblast proliferation via ERK-dependent CCL7 upregulation. PloS One 14, e0222559. doi: 10.1371/journal.pone.0222559, PMID: 31518371 PMC6743866

[B114] Lee B. Shin M. Park Y. Won S. Y. Cho K. S. (2021). Physical exercise-induced myokines in neurodegenerative diseases. Int. J. Mol. Sci. 22 (11), 5795 doi: 10.3390/ijms22115795, PMID: 34071457 PMC8198301

[B115] Lee D.-H. Sung S.-C. Hong K.-S. (2023). Effects of regular aerobic exercise interventions on decreased cerebral blood flow-induced mild cognitive impairment. Exercise Sci. 32, 242–254. doi: 10.15857/ksep.2023.00255

[B116] Leighton S. P. Nerurkar L. Krishnadas R. Johnman C. Graham G. J. Cavanagh J. (2018). Chemokines in depression in health and in inflammatory illness: a systematic review and meta-analysis. Mol. Psychiatry 23, 48–58. doi: 10.1038/mp.2017.205, PMID: 29133955 PMC5754468

[B117] Leuchtmann A. B. Adak V. Dilbaz S. Handschin C. (2021). The role of the skeletal muscle secretome in mediating endurance and resistance training adaptations. Front. Physiol. 12. doi: 10.3389/fphys.2021.709807, PMID: 34456749 PMC8387622

[B118] Libardi C. A. De Souza G. V. Cavaglieri C. R. Madruga V. A. Chacon-Mikahil M. P. T. (2012). Effect of resistance, endurance, and concurrent training on TNF-α, IL-6, and CRP. Med. Sci. Sports Exercise 44, 50–56. doi: 10.1249/MSS.0b013e318229d2e9, PMID: 21697747

[B119] Licinio J. Wong M. L. (1997). Pathways and mechanisms for cytokine signaling of the central nervous system. J. Clin. Invest. 100, 2941–2947. doi: 10.1172/Jci119846, PMID: 9399938 PMC508504

[B120] Lin W. Song H. Shen J. Wang J. Yang Y. Yang Y. . (2023). Functional role of skeletal muscle-derived interleukin-6 and its effects on lipid metabolism. Front. Physiol. 14. doi: 10.3389/fphys.2023.1110926, PMID: 37555019 PMC10405179

[B121] Liu Y. Chu J. M. T. Yan T. Zhang Y. Chen Y. Chang R. C. C. . (2020). Short-term resistance exercise inhibits neuroinflammation and attenuates neuropathological changes in 3xTg Alzheimer’s disease mice. J. Neuroinflamm. 17, 4. 10.1186/s12974-019-1653-7 PMC6942350 31900170

[B122] Liu M. Pan J. Li X. Zhang X. Tian F. Li M. . (2024). Interleukin-6 deficiency reduces neuroinflammation by inhibiting the STAT3-cGAS-STING pathway in Alzheimer’s disease mice. J. Neuroinflamm. 21, 282. doi: 10.1186/s12974-024-03277-3, PMID: 39487531 PMC11529443

[B123] Liu Fu Zhao X. Cui R. Yang W. (2024). The role of exercise-related FNDC5/irisin in depression. Front. Pharmacol. 15. doi: 10.3389/fphar.2024.1461995, PMID: 39484160 PMC11524886

[B124] Long J. M. Holtzman D. M. (2019). Alzheimer disease: an update on pathobiology and treatment strategies. Cell 179, 312–339. doi: 10.1016/j.cell.2019.09.001, PMID: 31564456 PMC6778042

[B125] López-Ortiz S. Lista S. Valenzuela P. L. Pinto-Fraga J. Carmona R. Caraci F. . (2023). Effects of physical activity and exercise interventions on Alzheimer’s disease: an umbrella review of existing meta-analyses. J. Neurol. 270, 711–725. doi: 10.1007/s00415-022-11454-8, PMID: 36342524

[B126] Lopez-Rodriguez A. B. Hennessy E. Murray C. L. Nazmi A. Delaney H. J. Healy D. . (2021). Acute systemic inflammation exacerbates neuroinflammation in Alzheimer’s disease: IL-1beta drives amplified responses in primed astrocytes and neuronal network dysfunction. Alzheimers Dementia 17, 1735–1755. doi: 10.1002/alz.12341, PMID: 34080771 PMC8874214

[B127] Lourenco M. V. Frozza R. L. de Freitas G. B. Zhang H. Kincheski G. C. Ribeiro F. C. . (2019). Exercise-linked FNDC5/irisin rescues synaptic plasticity and memory defects in Alzheimer’s models. Nat. Med. 25, 165–175. 30617325 10.1038/s41591-018-0275-4 PMC6327967

[B128] Lourenco M. V. Ribeiro F. C. Sudo F. K. Drummond C. Assuncao N. Vanderborght B. . (2020). Cerebrospinal fluid irisin correlates with amyloid-beta, BDNF, and cognition in Alzheimer’s disease. Alzheimers Dement (Amst) 12, e12034. doi: 10.1002/dad2.12034, PMID: 32582833 PMC7306518

[B129] Lu B. Nagappan G. Lu Y. (2014). BDNF and synaptic plasticity, cognitive function, and dysfunction. Handb. Exp. Pharmacol. 220, 223–250. doi: 10.1007/978-3-642-45106-5_9, PMID: 24668475

[B130] Ma Q. Tian J.-L. Lou Y. Guo R. Ma X.-R. Wu J.-B. . (2025). Oligodendrocytes drive neuroinflammation and neurodegeneration in Parkinson’s disease via the prosaposin-GPR37-IL-6 axis. Cell Rep. 44 (2), 115266 39913287 10.1016/j.celrep.2025.115266

[B131] Ma Z. Xu Y. Lian P. Wu Y. Liu K. Zhang Z. . (2025). Alpha-synuclein Fibrils Inhibit Activation of the BDNF/ERK Signaling Loop in the mPFC to Induce Parkinson’s Disease-like Alterations with Depression: Z. Ma et al.: PFF Inhibit BDNF/ERK in the mPFC to Induce PD Dep. Neurosci. Bull. 41, 951–969. 39609371 10.1007/s12264-024-01323-x PMC12158912

[B132] Maak S. Norheim F. Drevon C. A. Erickson H. P. (2021). Progress and challenges in the biology of FNDC5 and irisin. Endocr. Rev. 42, 436–456. doi: 10.1210/endrev/bnab003, PMID: 33493316 PMC8284618

[B133] Mao Q. S. Guo Y. X. Tian X. L. Zhao H. L. Kong Y. Z. (2025). Global burden of mental disorders in 204 countries and territories results from the Global Burden of Disease Study 2021. World J. Psychiatry 15, 106887. doi: 10.5498/wjp.v15.i8.106887, PMID: 40837810 PMC12362664

[B134] Marano L. Missaglia S. Martegani E. Bonanomi A. Tremolada C. Tavian D. . (2025). Plasma and salivary irisin response to moderate load/high volume resistance exercise in young, resistance-trained men. Eur. J. Transl. Myol 35 (3), 13957 doi: 10.4081/ejtm.2025.13957, PMID: 40631960 PMC12536678

[B135] Maren S. Holmes A. (2016). Stress and fear extinction. Neuropsychopharmacology 41, 58–79. doi: 10.1038/npp.2015.180, PMID: 26105142 PMC4677122

[B136] Marston K. J. Newton M. J. Brown B. M. Rainey-Smith S. R. Bird S. Martins R. N. . (2017). Intense resistance exercise increases peripheral brain-derived neurotrophic factor. J. Sci. Med. Sport 20, 899–903. doi: 10.1016/j.jsams.2017.03.015, PMID: 28511848

[B137] Matsuda S. Fujita T. Kajiya M. Kashiwai K. Takeda K. Shiba H. . (2015). Brain-derived neurotrophic factor prevents the endothelial barrier dysfunction induced by interleukin-1beta and tumor necrosis factor-alpha. J. Periodontal Res. 50, 444–451. doi: 10.1111/jre.12226, PMID: 25203938

[B138] Matthews V. B. Astrom M. B. Chan M. H. Bruce C. R. Krabbe K. S. Prelovsek O. . (2009). Brain-derived neurotrophic factor is produced by skeletal muscle cells in response to contraction and enhances fat oxidation via activation of AMP-activated protein kinase. Diabetologia 52, 1409–1418. doi: 10.1007/s00125-009-1364-1, PMID: 19387610

[B139] Mazo C. E. Miranda E. R. Shadiow J. Vesia M. Haus J. M. (2022). High intensity acute aerobic exercise elicits alterations in circulating and skeletal muscle tissue expression of neuroprotective exerkines. Brain Plasticity 8, 5–18. doi: 10.3233/BPL-220137, PMID: 36448040 PMC9661358

[B140] Mee-Inta O. Zhao Z. W. Kuo Y. M. (2019). Physical exercise inhibits inflammation and microglial activation. Cells 8 (7), 691 doi: 10.3390/cells8070691, PMID: 31324021 PMC6678635

[B141] Mehdi S. Wani S. U. D. Krishna K. L. Kinattingal N. Roohi T. F. (2023). A review on linking stress, depression, and insulin resistance via low-grade chronic inflammation. Biochem. Biophys. Rep. 36, 101571. doi: 10.1016/j.bbrep.2023.101571, PMID: 37965066 PMC10641573

[B142] Menard C. Pfau M. L. Hodes G. E. Kana V. Wang V. X. Bouchard S. . (2017). Social stress induces neurovascular pathology promoting depression. Nat. Neurosci. 20, 1752–1760. doi: 10.1038/s41593-017-0010-3, PMID: 29184215 PMC5726568

[B143] Michell B. J. Griffiths J. E. Mitchelhill K. I. Rodriguez-Crespo I. Tiganis T. Bozinovski S. . (1999). The Akt kinase signals directly to endothelial nitric oxide synthase. Curr. Biol. 9, 845–848. doi: 10.1016/s0960-9822(99)80371-6, PMID: 10469573

[B144] Middelbeek R. J. W. Motiani P. Brandt N. Nigro P. Zheng J. Virtanen K. A. . (2021). Exercise intensity regulates cytokine and klotho responses in men. Nutr. Diabetes 11, 5. doi: 10.1038/s41387-020-00144-x, PMID: 33414377 PMC7791135

[B145] Miller A. H. (2025). Advancing an inflammatory subtype of major depression. Am. J. Psychiatry 182, 516–524. doi: 10.1176/appi.ajp.20250289, PMID: 40329642 PMC12282100

[B146] Mirjalili R. Shokouh E. Dehkordi N. S. Afsari R. Shafia S. Rashidy-Pour A. (2022). Prior short-term exercise prevents behavioral and biochemical abnormalities induced by single prolonged stress in a rat model of posttraumatic stress disorder. Behav. Brain Res. 428, 113864. doi: 10.1016/j.bbr.2022.113864, PMID: 35405172

[B147] Mitchell A. J. Cogswell I. E. Dalos J. Tsakalos G. Lei J. Oros A. . (2025). Estimating global direct health-care spending on neurological and mental health between 2000 and 2019: a modelling study. Lancet Public Health 10, e401–e411. doi: 10.1016/S2468-2667(25)00089-1, PMID: 40312084

[B148] Mojtabavi H. Saghazadeh A. van den Heuvel L. Bucker J. Rezaei N. (2020). Peripheral blood levels of brain-derived neurotrophic factor in patients with post-traumatic stress disorder (PTSD): A systematic review and meta-analysis. PloS One 15, e0241928. doi: 10.1371/journal.pone.0241928, PMID: 33152026 PMC7644072

[B149] Montgomery T. R. Jr. Grant D. M. (2026). Neurobiological, molecular, and systemic mechanisms of exercise in the treatment of mental health disorders. J. Psychiatr. Res. 195, 113–122. doi: 10.1016/j.jpsychires.2026.01.043, PMID: 41619687

[B150] Moon H. Y. Becke A. Berron D. Becker B. Sah N. Benoni G. . (2016). Running-induced systemic cathepsin B secretion is associated with memory function. Cell Metab. 24, 332–340. doi: 10.1016/j.cmet.2016.05.025, PMID: 27345423 PMC6029441

[B151] Musaro A. McCullagh K. Paul A. Houghton L. Dobrowolny G. Molinaro M. . (2001). Localized Igf-1 transgene expression sustains hypertrophy and regeneration in senescent skeletal muscle. Nat. Genet. 27, 195–200. doi: 10.1038/84839, PMID: 11175789

[B152] Nagamatsu L. S. Flicker L. Kramer A. F. Voss M. W. Erickson K. I. Hsu C. L. . (2014). Exercise is medicine, for the body and the brain. Br. J. Sports Med. 48(12), 943–944. doi: 10.1136/bjsports-2013-093224, PMID: 24659507 PMC4330095

[B153] Nakanishi H. (2020). Microglial cathepsin B as a key driver of inflammatory brain diseases and brain aging. Neural Regener. Res. 15, 25–29. doi: 10.4103/1673-5374.264444, PMID: 31535638 PMC6862407

[B154] Ni J. J. Lan F. Xu Y. Nakanishi H. Li X. (2022). Extralysosomal cathepsin B in central nervous system: Mechanisms and therapeutic implications. Brain Pathol. 32 (5) e13071 doi: 10.1111/bpa.13071, PMID: 35411983 PMC9425006

[B155] Nicolini C. Toepp S. Harasym D. Michalski B. Fahnestock M. Gibala M. J. . (2019). No changes in corticospinal excitability, biochemical markers, and working memory after six weeks of high-intensity interval training in sedentary males. Physiol. Rep. 7, e14140. doi: 10.14814/phy2.14140, PMID: 31175708 PMC6555846

[B156] Nie Y. Liu D. (2017). N-Glycosylation is required for FDNC5 stabilization and irisin secretion. Biochem. J. 474, 3167–3177. doi: 10.1042/BCJ20170241, PMID: 28733331

[B157] Nieman D. C. Davis J. M. Henson D. A. Walberg-Rankin J. Shute M. Dumke C. L. . (2003). Carbohydrate ingestion influences skeletal muscle cytokine mRNA and plasma cytokine levels after a 3-h run. J. Appl. Physiol. 94, 1917–1925. doi: 10.1152/japplphysiol.01130.2002, PMID: 12533503

[B158] Nieto-Estévez V. Defterali Ç. Vicario-Abejón C. (2016). IGF-I: A key growth factor that regulates neurogenesis and synaptogenesis from embryonic to adult stages of the brain. Front. Neurosci. 10. doi: 10.3389/fnins.2016.00052, PMID: 26941597 PMC4763060

[B159] Nindl B. C. Alemany J. A. Tuckow A. P. Kellogg M. D. Sharp M. A. Patton J. F. (2009). Effects of exercise mode and duration on 24-h IGF-I system recovery responses. Med. Sci. sports Exercise 41, 1261–1270. doi: 10.1249/MSS.0b013e318197125c, PMID: 19461539

[B160] Nygaard H. Slettalokken G. Vegge G. Hollan I. Whist J. E. Strand T. . (2015). Irisin in blood increases transiently after single sessions of intense endurance exercise and heavy strength training. PloS One 10, e0121367. doi: 10.1371/journal.pone.0121367, PMID: 25781950 PMC4363689

[B161] Oliveira C. L. P. de Moura Mello Antunes B. Gomes A. C. Lira F. S. Pimentel G. D. Boulé N. G. . (2020). Creatine supplementation does not promote additional effects on inflammation and insulin resistance in older adults: A pilot randomized, double-blind, placebo-controlled trial. Clin. Nutr. ESPEN 38, 94–98. doi: 10.1016/j.clnesp.2020.05.024, PMID: 32690185

[B162] Ortenlof N. Vallius S. Karlsson H. Ekstrom C. Kristiansson A. Holmqvist B. . (2024). Choroid plexus extracellular vesicle transport of blood-borne insulin-like growth factor 1 to the hippocampus of the immature brain. Pnas Nexus 3, pgae496. doi: 10.1093/pnasnexus/pgae496, PMID: 39660059 PMC11630522

[B163] Pabon M. M. Bachstetter A. D. Hudson C. E. Gemma C. Bickford P. C. (2011). CX3CL1 reduces neurotoxicity and microglial activation in a rat model of Parkinson’s disease. J. Neuroinflamm. 8, 9. doi: 10.1186/1742-2094-8-9, PMID: 21266082 PMC3039584

[B164] Pan W. H. Banks W. A. Fasold M. B. Bluth J. Kastin A. J. (1998). Transport of brain-derived neurotrophic factor across the blood-brain barrier. Neuropharmacology 37, 1553–1561. doi: 10.1016/S0028-3908(98)00141-5, PMID: 9886678

[B165] Pan W. H. Kastin A. J. (2000). Interactions of IGF-1 with the blood-brain barrier *in vivo* and in situ. Neuroendocrinology 72, 171–178. doi: 10.1159/000054584, PMID: 11025411

[B166] Pang Y. Zheng B. Campbell L. R. Fan L. W. Cai Z. Rhodes P. G. (2010). IGF-1 can either protect against or increase LPS-induced damage in the developing rat brain. Pediatr. Res. 67, 579–584. doi: 10.1203/PDR.0b013e3181dc240f, PMID: 20220546 PMC3076081

[B167] Pardridge W. M. (2007). Blood-brain barrier delivery of protein and non-viral gene therapeutics with molecular Trojan horses. J. Control Release 122, 345–348. doi: 10.1016/j.jconrel.2007.04.001, PMID: 17512078 PMC2701689

[B168] Passos I. C. Vasconcelos-Moreno M. P. Costa L. G. Kunz M. Brietzke E. Quevedo J. . (2015). Inflammatory markers in post-traumatic stress disorder: a systematic review, meta-analysis, and meta-regression. Lancet Psychiatry 2, 1002–1012. doi: 10.1016/S2215-0366(15)00309-0, PMID: 26544749

[B169] Pedersen B. K. (2019). Physical activity and muscle-brain crosstalk. Nat. Rev. Endocrinol. 15, 383–392. doi: 10.1038/s41574-019-0174-x, PMID: 30837717

[B170] Pedersen B. K. (2023). From the discovery of myokines to exercise as medicine. Dan Med. J. 32 (5) e13071 37622635

[B171] Pedersen B. K. Febbraio M. A. (2008). Muscle as an endocrine organ: focus on muscle-derived interleukin-6. Physiol. Rev. 88, 1379–1406. doi: 10.1152/physrev.90100.2007, PMID: 18923185

[B172] Pedersen B. K. Febbraio M. A. (2012). Muscles, exercise and obesity: skeletal muscle as a secretory organ. Nat. Rev. Endocrinol. 8, 457–465. doi: 10.1038/nrendo.2012.49, PMID: 22473333

[B173] Pena G. S. Paez H. G. Johnson T. K. Halle J. L. Carzoli J. P. Visavadiya N. P. . (2020). Hippocampal growth factor and myokine cathepsin B expression following aerobic and resistance training in 3xTg-AD mice. Int. J. chronic Dis. 2020, 5919501. 32090058 10.1155/2020/5919501 PMC7011393

[B174] Petersen A. M. Pedersen B. K. (2005). The anti-inflammatory effect of exercise. J. Appl. Physiol. (1985) 98, 1154–1162. doi: 10.1152/japplphysiol.00164.2004, PMID: 15772055

[B175] Philippou A. Maridaki M. Pneumaticos S. Koutsilieris M. (2014). The complexity of the IGF1 gene splicing, posttranslational modification and bioactivity. Mol. Med. 20, 202–214. doi: 10.2119/molmed.2014.00011, PMID: 24637928 PMC4022784

[B176] Pruunsild P. Kazantseva A. Aid T. Palm K. Timmusk T. (2007). Dissecting the human BDNF locus: bidirectional transcription, complex splicing, and multiple promoters. Genomics 90, 397–406. doi: 10.1016/j.ygeno.2007.05.004, PMID: 17629449 PMC2568880

[B177] Puntambekar S. S. Moutinho M. Lin P. B. Jadhav V. Tumbleson-Brink D. Balaji A. . (2022). CX3CR1 deficiency aggravates amyloid driven neuronal pathology and cognitive decline in Alzheimer’s disease. Mol. Neurodegener. 17, 47. doi: 10.1186/s13024-022-00545-9, PMID: 35764973 PMC9241248

[B178] Qaisar R. Bhaskaran S. Van Remmen H. (2016). Muscle fiber type diversification during exercise and regeneration. Free Radic. Biol. Med. 98, 56–67. doi: 10.1016/j.freeradbiomed.2016.03.025, PMID: 27032709

[B179] Qu Y. Li J. Qin Q. Wang D. Zhao J. An K. . (2023). A systematic review and meta-analysis of inflammatory biomarkers in Parkinson’s disease. NPJ Parkinsons Dis. 9, 18. doi: 10.1038/s41531-023-00449-5, PMID: 36739284 PMC9899271

[B180] Rai M. Demontis F. (2022). Muscle-to-brain signaling via myokines and myometabolites. Brain Plast. 8, 43–63. doi: 10.3233/BPL-210133, PMID: 36448045 PMC9661353

[B181] Reza M. M. Subramaniyam N. Sim C. M. Ge X. Sathiakumar D. McFarlane C. . (2017). Irisin is a pro-myogenic factor that induces skeletal muscle hypertrophy and rescues denervation-induced atrophy. Nat. Commun. 8, 1104. doi: 10.1038/s41467-017-01131-0, PMID: 29062100 PMC5653663

[B182] Rhea E. M. Rask-Madsen C. Banks W. A. (2018). Insulin transport across the blood-brain barrier can occur independently of the insulin receptor. J. Physiol. 596, 4753–4765. doi: 10.1113/JP276149, PMID: 30044494 PMC6166047

[B183] Ritson M. Wheeler-Jones C. P. D. Stolp H. B. (2024). Endothelial dysfunction in neurodegenerative disease: Is endothelial inflammation an overlooked druggable target? J. Neuroimmunol 391, 578363. doi: 10.1016/j.jneuroim.2024.578363, PMID: 38728929

[B184] Rose-John S. (2012). IL-6 trans-signaling via the soluble IL-6 receptor: importance for the pro-inflammatory activities of IL-6. Int. J. Biol. Sci. 8, 1237–1247. doi: 10.7150/ijbs.4989, PMID: 23136552 PMC3491447

[B185] Rotwein P. (2012). Mapping the growth hormone--Stat5b--IGF-I transcriptional circuit. Trends Endocrinol. Metab. 23, 186–193. doi: 10.1016/j.tem.2012.01.001, PMID: 22361342 PMC3313013

[B186] Ruiz-Gonzalez D. Hernandez-Martinez A. Valenzuela P. L. Morales J. S. Soriano-Maldonado A. (2021). Effects of physical exercise on plasma brain-derived neurotrophic factor in neurodegenerative disorders: A systematic review and meta-analysis of randomized controlled trials. Neurosci. Biobehav. Rev. 128, 394–405. doi: 10.1016/j.neubiorev.2021.05.025, PMID: 34087277

[B187] Safdar A. Saleem A. Tarnopolsky M. A. (2016). The potential of endurance exercise-derived exosomes to treat metabolic diseases. Nat. Rev. Endocrinol. 12, 504–517. doi: 10.1038/nrendo.2016.76, PMID: 27230949

[B188] Sah A. Singewald N. (2025). The (neuro)inflammatory system in anxiety disorders and PTSD: Potential treatment targets. Pharmacol. Ther. 269, 108825. doi: 10.1016/j.pharmthera.2025.108825, PMID: 39983845

[B189] Sandri M. Sandri C. Gilbert A. Skurk C. Calabria E. Picard A. . (2004). Foxo transcription factors induce the atrophy-related ubiquitin ligase atrogin-1 and cause skeletal muscle atrophy. Cell 117, 399–412. doi: 10.1016/s0092-8674(04)00400-3, PMID: 15109499 PMC3619734

[B190] Saudenova M. Promnitz J. Ohrenschall G. Himmerkus N. Bottner M. Kunke M. . (2022). Behind every smile there’s teeth: Cathepsin B’s function in health and disease with a kidney view. Biochim. Biophys. Acta Mol. Cell Res. 1869, 119190. doi: 10.1016/j.bbamcr.2021.119190, PMID: 34968578

[B191] Schiaffino S. Mammucari C. (2011). Regulation of skeletal muscle growth by the IGF1-Akt/PKB pathway: insights from genetic models. Skelet Muscle 1, 4. doi: 10.1186/2044-5040-1-4, PMID: 21798082 PMC3143906

[B192] Schwarz A. J. Brasel J. A. Hintz R. L. Mohan S. Cooper D. M. (1996). Acute effect of brief low- and high-intensity exercise on circulating insulin-like growth factor (IGF) I, II, and IGF-binding protein-3 and its proteolysis in young healthy men. J. Clin. Endocrinol. Metab. 81, 3492–3497. doi: 10.1210/jcem.81.10.8855791, PMID: 8855791

[B193] Severinsen M. C. K. Pedersen B. K. (2020). Muscle–organ crosstalk: the emerging roles of myokines. Endocrine Rev. 41, 594–609. 32393961 10.1210/endrev/bnaa016 PMC7288608

[B194] Shan T. Liang X. Bi P. Kuang S. (2013). Myostatin knockout drives browning of white adipose tissue through activating the AMPK-PGC1alpha-Fndc5 pathway in muscle. FASEB J. 27, 1981–1989. doi: 10.1096/fj.12-225755, PMID: 23362117 PMC3633817

[B195] Shan W. P. Yan S. L. Guo Y. Y. Yang H. K. Wang J. C. Xiang J. (2025). Aerobic exercise and gut flora: a key link to improved cognitive impairment in mice with Parkinson’s disease. Front. Aging Neurosci. 17. doi: 10.3389/fnagi.2025.1630003, PMID: 41195080 PMC12583108

[B196] Shanks H. R. C. Chen K. Reiman E. M. Blennow K. Cummings J. L. Massa S. M. . (2024). p75 neurotrophin receptor modulation in mild to moderate Alzheimer disease: a randomized, placebo-controlled phase 2a trial. Nat. Med. 30, 1761–1770. doi: 10.1038/s41591-024-02977-w, PMID: 38760589 PMC11186782

[B197] Sheridan G. K. Murphy K. J. (2013). Neuron-glia crosstalk in health and disease: fractalkine and CX3CR1 take centre stage. Open Biol. 3, 130181. doi: 10.1098/rsob.130181, PMID: 24352739 PMC3877844

[B198] Shi H. Hao X. Sun Y. Zhao Y. Wang Y. Cao X. . (2024). Exercise-inducible circulating extracellular vesicle irisin promotes browning and the thermogenic program in white adipose tissue. Acta Physiol. (Oxf) 240, e14103. doi: 10.1111/apha.14103, PMID: 38288566

[B199] Shin J. W. An S. Kim D. Kim H. Ahn J. Eom J. . (2022). An IGF1 receptor-based shuttle, mediates efficient delivery of biologics across the blood-brain barrier. Cell Rep. Methods 2, 100338. doi: 10.1016/j.crmeth.2022.100338, PMID: 36452865 PMC9701613

[B200] Siddiqui A. A. Merquiol E. Bruck-Haimson R. Hirbawi J. Boocholez H. Cohen I. . (2024). Cathepsin B promotes Abeta proteotoxicity by modulating aging regulating mechanisms. Nat. Commun. 15, 8564. doi: 10.1038/s41467-024-52540-x, PMID: 39362844 PMC11450018

[B201] Singh S. Anshita D. Ravichandiran V. (2021). MCP-1: Function, regulation, and involvement in disease. Int. Immunopharmacol 101, 107598. doi: 10.1016/j.intimp.2021.107598, PMID: 34233864 PMC8135227

[B202] Steensberg A. van Hall G. Osada T. Sacchetti M. Saltin B. Klarlund Pedersen B. (2000). Production of interleukin-6 in contracting human skeletal muscles can account for the exercise-induced increase in plasma interleukin-6. J. Physiol. 529, 237–242. doi: 10.1111/j.1469-7793.2000.00237.x, PMID: 11080265 PMC2270169

[B203] Ströhle A. Stoy M. Graetz B. Scheel M. Wittmann A. Gallinat J. . (2010). Acute exercise ameliorates reduced brain-derived neurotrophic factor in patients with panic disorder. Psychoneuroendocrinology 35, 364–368. 19682803 10.1016/j.psyneuen.2009.07.013

[B204] Strohm A. O. Majewska A. K. (2024). Physical exercise regulates microglia in health and disease. Front. Neurosci. 18. doi: 10.3389/fnins.2024.1420322, PMID: 38911597 PMC11192042

[B205] Strömberg A Olsson K Dijksterhuis JP Rullman E Schulte G Gustafsson T (2016). CX3CL1--a macrophage chemoattractant induced by a single bout of exercise in human skeletal muscle. Am J Physiol Regul Integr Comp Physiol. 310 (3), R297–304. doi: 10.1152/ajpregu.00236.2015, PMID: 26632602

[B206] Sugimoto T. Nakamura T. Yokoyama S. Fujisato T. Konishi S. Hashimoto T. (2022). Investigation of brain function-related myokine secretion by using contractile 3D-engineered muscle. Int. J. Mol. Sci. 23 (10) 5723 doi: 10.3390/ijms23105723, PMID: 35628536 PMC9144730

[B207] Sukhanov S. Higashi Y. Shai S. Y. Vaughn C. Mohler J. Li Y. . (2007). IGF-1 reduces inflammatory responses, suppresses oxidative stress, and decreases atherosclerosis progression in ApoE-deficient mice. Arterioscler. Thromb. Vasc. Biol. 27, 2684–2690. doi: 10.1161/ATVBAHA.107.156257, PMID: 17916769

[B208] Sung K.-Y. Kang S. Park J. Y. Park K. M. (2017). Effects of myokine factors on exercise types in obese women. Exercise Sci. 26, 275–280. doi: 10.15857/ksep.2017.26.4.275

[B209] Swalsingh G. Pani P. Sadayappan S. Bal N. C. (2025). Fractalkine is a key player in skeletal muscle metabolism and pathophysiology. FEBS J. 10.1111/febs.70267 40995677

[B210] Sweeney M. D. Sagare A. P. Zlokovic B. V. (2018). Blood-brain barrier breakdown in Alzheimer disease and other neurodegenerative disorders. Nat. Rev. Neurol. 14, 133–150. doi: 10.1038/nrneurol.2017.188, PMID: 29377008 PMC5829048

[B211] Szymura J. Kubica J. Wiecek M. Pera J. (2020). The immunomodulary effects of systematic exercise in older adults and people with parkinson’s disease. J. Clin. Med. 9 (1) 184 doi: 10.3390/jcm9010184, PMID: 31936624 PMC7019419

[B212] Taddei A. Giampietro C. Conti A. Orsenigo F. Breviario F. Pirazzoli V. . (2008). Endothelial adherens junctions control tight junctions by VE-cadherin-mediated upregulation of claudin-5. Nat. Cell Biol. 10, 923–934. doi: 10.1038/ncb1752, PMID: 18604199

[B213] Takata F. Nakagawa S. Matsumoto J. Dohgu S. (2021). Blood-brain barrier dysfunction amplifies the development of neuroinflammation: understanding of cellular events in brain microvascular endothelial cells for prevention and treatment of BBB dysfunction. Front. Cell. Neurosci. 15. doi: 10.3389/fncel.2021.661838, PMID: 34588955 PMC8475767

[B214] Takei Y. Amagase Y. Iida K. Sagawa T. Goto A. Kambayashi R. . (2022). Alteration in peritoneal cells with the chemokine CX3CL1 reverses age-associated impairment of recognition memory. Geroscience 44, 2305–2318. doi: 10.1007/s11357-022-00579-3, PMID: 35593945 PMC9616991

[B215] Tang C. Liu M. Zhou Z. Li H. Yang C. Yang L. . (2023). Treadmill exercise alleviates cognition disorder by activating the FNDC5: dual role of integrin alphaV/beta5 in parkinson’s disease. Int. J. Mol. Sci. 24 (9) 7830 doi: 10.3390/ijms24097830, PMID: 37175535 PMC10178565

[B216] Threlkeld S. W. Lynch J. L. Lynch K. M. Sadowska G. B. Banks W. A. Stonestreet B. S. (2010). Ovine proinflammatory cytokines cross the murine blood-brain barrier by a common saturable transport mechanism. Neuroimmunomodulation 17, 405–410. doi: 10.1159/000288265, PMID: 20516722 PMC2914440

[B217] Tozzi L. Zhang X. Pines A. Olmsted A. M. Zhai E. S. Anene E. T. . (2024). Personalized brain circuit scores identify clinically distinct biotypes in depression and anxiety. Nat. Med. 30, 2076–2087. doi: 10.1038/s41591-024-03057-9, PMID: 38886626 PMC11271415

[B218] Trejo J. L. Carro E. Torres-Aleman I. (2001). Circulating insulin-like growth factor I mediates exercise-induced increases in the number of new neurons in the adult hippocampus. J. Neurosci. 21, 1628–1634. doi: 10.1523/JNEUROSCI.21-05-01628.2001, PMID: 11222653 PMC6762955

[B219] Tsubokawa T. Solaroglu I. Yatsushige H. Cahill J. Yata K. Zhang J. H. (2006). Cathepsin and calpain inhibitor E64d attenuates matrix metalloproteinase-9 activity after focal cerebral ischemia in rats. Stroke 37, 1888–1894. doi: 10.1161/01.STR.0000227259.15506.24, PMID: 16763180

[B220] Tsuchiya Y. Ando D. Goto K. Kiuchi M. Yamakita M. Koyama K. (2014). High-intensity exercise causes greater irisin response compared with low-intensity exercise under similar energy consumption. Tohoku J. Exp. Med. 233, 135–140. doi: 10.1620/tjem.233.135, PMID: 24910199

[B221] Tsuchiya Y. Ando D. Takamatsu K. Goto K. (2015). Resistance exercise induces a greater irisin response than endurance exercise. Metabolism 64, 1042–1050. doi: 10.1016/j.metabol.2015.05.010, PMID: 26081427

[B222] Turk V. Stoka V. Vasiljeva O. Renko M. Sun T. Turk B. . (2012). Cysteine cathepsins: from structure, function and regulation to new frontiers. Biochim. Biophys. Acta 1824, 68–88. doi: 10.1016/j.bbapap.2011.10.002, PMID: 22024571 PMC7105208

[B223] Vaughan R. A. Gannon N. P. Barberena M. A. Garcia-Smith R. Bisoffi M. Mermier C. M. . (2014). Characterization of the metabolic effects of irisin on skeletal muscle in *vitro*. Diabetes Obes. Metab. 16, 711–718. doi: 10.1111/dom.12268, PMID: 24476050

[B224] Verboogen D. R. J. Revelo N. H. Ter Beest M. van den Bogaart G. (2019). Interleukin-6 secretion is limited by self-signaling in endosomes. J. Mol. Cell Biol. 11, 144–157. doi: 10.1093/jmcb/mjy038, PMID: 30016456 PMC6392102

[B225] Verheggen R. J. H. M. Poelkens F. Roerink S. H. P. P. Ramakers R. E. F. S. Catoire M. Hermus A. R. M. M. . (2016). Exercise improves insulin sensitivity in the absence of changes in cytokines. Med. Sci. Sports Exercise 48, 2378–2386. doi: 10.1249/MSS.0000000000001035, PMID: 27414688

[B226] Wang B. Li N. Wang Y. Tian X. Lin J. Zhang X. . (2025). Exercise ameliorates dopaminergic neurodegeneration in parkinson’s disease mice by suppressing microglia-regulated neuroinflammation through irisin/AMPK/sirt1 pathway. Biol. (Basel) 14. (8) 955 doi: 10.3390/biology14080955, PMID: 40906143 PMC12383941

[B227] Wang T. Swaminathan S. K. Kandimalla K. K. Kalari K. R. (2022). Mapping the dynamics of insulin-responsive pathways in the blood-brain barrier endothelium using time-series transcriptomics data. NPJ Syst. Biol. Appl. 8, 29. doi: 10.1038/s41540-022-00235-8, PMID: 35974022 PMC9381797

[B228] Wang Y. Tian M. Tan J. Pei X. Lu C. Xin Y. . (2022). Irisin ameliorates neuroinflammation and neuronal apoptosis through integrin alphaVbeta5/AMPK signaling pathway after intracerebral hemorrhage in mice. J. Neuroinflamm. 19, 82. doi: 10.1186/s12974-022-02438-6, PMID: 35392928 PMC8988353

[B229] Wang X. W. Yuan L. J. Yang Y. Zhang M. Chen W. F. (2020). IGF-1 inhibits MPTP/MPP(+)-induced autophagy on dopaminergic neurons through the IGF-1R/PI3K-Akt-mTOR pathway and GPER. Am. J. Physiol. Endocrinol. Metab. 319, E734–E743. doi: 10.1152/ajpendo.00071.2020, PMID: 32865008

[B230] Whitham M. Chan M. H. Pal M. Matthews V. B. Prelovsek O. Lunke S. . (2012). Contraction-induced interleukin-6 gene transcription in skeletal muscle is regulated by c-Jun terminal kinase/activator protein-1. J. Biol. Chem. 287, 10771–10779. doi: 10.1074/jbc.M111.310581, PMID: 22351769 PMC3322851

[B231] Whitham M. Parker B. L. Friedrichsen M. Hingst J. R. Hjorth M. Hughes W. E. . (2018). Extracellular vesicles provide a means for tissue crosstalk during exercise. Cell Metab. 27, 237–251 e234. doi: 10.1016/j.cmet.2017.12.001, PMID: 29320704

[B232] Witmer N. H. Linzer C. R. Boudreau R. L. (2024). Fndc5 is translated from an upstream ATG start codon and cleaved to produce irisin myokine precursor protein in humans and mice. Cell Metab. 36, 879–881. doi: 10.1016/j.cmet.2024.02.008, PMID: 38471509 PMC11555857

[B233] Wrann C. D. (2015). FNDC5/irisin - their role in the nervous system and as a mediator for beneficial effects of exercise on the brain. Brain Plast. 1, 55–61. doi: 10.3233/BPL-150019, PMID: 28480165 PMC5419585

[B234] Wrann C. D. White J. P. Salogiannnis J. Laznik-Bogoslavski D. Wu J. Ma D. . (2013). Exercise induces hippocampal BDNF through a PGC-1α/FNDC5 pathway. Cell Metab. 18, 649–659. doi: 10.1016/j.cmet.2013.09.008, PMID: 24120943 PMC3980968

[B235] Wu D. Pardridge W. M. (1999). Neuroprotection with noninvasive neurotrophin delivery to the brain. Proc. Natl. Acad. Sci. U.S.A. 96, 254–259. doi: 10.1073/pnas.96.1.254, PMID: 9874805 PMC15126

[B236] Xiao K. Luo Y. Liang X. Tang J. Wang J. Xiao Q. . (2021). Beneficial effects of running exercise on hippocampal microglia and neuroinflammation in chronic unpredictable stress-induced depression model rats. Transl. Psychiatry 11, 461. doi: 10.1038/s41398-021-01571-9, PMID: 34489395 PMC8421357

[B237] Xie X. Ding Y. Yan Q. Zhao J. Zhang L. (2025). Irisin alleviates anxiety and deficits in fear extinction in PTSD within SPS mouse model. Neuropeptides 113, 102537. doi: 10.1016/j.npep.2025.102537, PMID: 40675031

[B238] Xie Z. Zhao M. Yan C. Kong W. Lan F. Zhao S. . (2023). Cathepsin B in programmed cell death machinery: mechanisms of execution and regulatory pathways. Cell Death Dis. 14, 255. doi: 10.1038/s41419-023-05786-0, PMID: 37031185 PMC10082344

[B239] Yoshida T. Semprun-Prieto L. Sukhanov S. Delafontaine P. (2010). IGF-1 prevents ANG II-induced skeletal muscle atrophy via Akt- and Foxo-dependent inhibition of the ubiquitin ligase atrogin-1 expression. Am. J. Physiol. Heart Circ. Physiol. 298, H1565–H1570. doi: 10.1152/ajpheart.00146.2010, PMID: 20228261 PMC2867436

[B240] Zaccari B. Higgins M. Haywood T. N. Patel M. Emerson D. Hubbard K. . (2023). Yoga vs cognitive processing therapy for military sexual trauma-related posttraumatic stress disorder: A randomized clinical trial. JAMA Netw. Open 6, e2344862. doi: 10.1001/jamanetworkopen.2023.44862, PMID: 38064219 PMC10709771

[B241] Zanou N. Gailly P. (2013). Skeletal muscle hypertrophy and regeneration: interplay between the myogenic regulatory factors (MRFs) and insulin-like growth factors (IGFs) pathways. Cell Mol. Life Sci. 70, 4117–4130. doi: 10.1007/s00018-013-1330-4, PMID: 23552962 PMC11113627

[B242] Zegeye M. M. Lindkvist M. Falker K. Kumawat A. K. Paramel G. Grenegard M. . (2018). Activation of the JAK/STAT3 and PI3K/AKT pathways are crucial for IL-6 trans-signaling-mediated pro-inflammatory response in human vascular endothelial cells. Cell Commun. Signal 16, 55. doi: 10.1186/s12964-018-0268-4, PMID: 30185178 PMC6125866

[B243] Zeng Y. Chourpiliadis C. Hammar N. Seitz C. Valdimarsdottir U. A. Fang F. . (2024). Inflammatory biomarkers and risk of psychiatric disorders. JAMA Psychiatry 81, 1118–1129. doi: 10.1001/jamapsychiatry.2024.2185, PMID: 39167384 PMC11339698

[B244] Zhang J. He Y. Yang P. Zhang H. Tong Y. Jiang L. . (2026). Microglial rack1 deficiency alleviates alzheimer’s disease pathology through enhancing IGF1-mediated astrocytic phagocytosis. Adv. Sci. (Weinh) 13, e15877. doi: 10.1002/advs.202515877, PMID: 41168999 PMC12806348

[B245] Zhang L. Hu X. Z. Li X. Chen Z. Benedek D. M. Fullerton C. S. . (2020). Potential chemokine biomarkers associated with PTSD onset, risk and resilience as well as stress responses in US military service members. Transl. Psychiatry 10, 31. doi: 10.1038/s41398-020-0693-1, PMID: 32066664 PMC7026448

[B246] Zhang Q. Xiang S. Chen X. Rong Y. Huang L. Chen Z. . (2024). Irisin attenuates acute glaucoma-induced neuroinflammation by activating microglia-integrin alphaVbeta5/AMPK and promoting autophagy. Int. Immunopharmacol 138, 112545. doi: 10.1016/j.intimp.2024.112545, PMID: 38955026

[B247] Zhang X. Xu S. Hu Y. Liu Q. Liu C. Chai H. . (2023). Irisin exhibits neuroprotection by preventing mitochondrial damage in Parkinson’s disease. NPJ Parkinsons Dis. 9, 13. doi: 10.1038/s41531-023-00453-9, PMID: 36720890 PMC9889817

[B248] Zhao R. (2022). Irisin at the crossroads of inter-organ communications: Challenge and implications. Front. Endocrinol. (Lausanne) 13. doi: 10.3389/fendo.2022.989135, PMID: 36267573 PMC9578559

[B249] Zhao J. Su Z. Qu C. Dong Y. (2017). Effects of 12 weeks resistance training on serum irisin in older male adults. Front. Physiol. 8. doi: 10.3389/fphys.2017.00171, PMID: 28382004 PMC5360699

[B250] Zhao R. Tian X. Xu H. Wang Y. Lin J. Wang B. (2025). Aerobic exercise restores hippocampal neurogenesis and cognitive function by decreasing microglia inflammasome formation through irisin/NLRP3 pathway. Aging Cell 24, e70061. doi: 10.1111/acel.70061, PMID: 40192010 PMC12266781

[B251] Zhen K. Zhang S. Y. Tao X. F. Li G. Lv Y. Y. Yu L. K. (2022). A systematic review and meta-analysis on effects of aerobic exercise in people with Parkinson’s disease. NPJ Parkinsons Dis. 8. (1) 146 doi: 10.1038/s41531-022-00418-4, PMID: 36316416 PMC9622812

